# Parkinson’s Disease–Associated LRRK2 Interferes with Astrocyte-Mediated Alpha-Synuclein Clearance

**DOI:** 10.1007/s12035-021-02327-8

**Published:** 2021-02-24

**Authors:** Linn Streubel-Gallasch, Veronica Giusti, Michele Sandre, Isabella Tessari, Nicoletta Plotegher, Elena Giusto, Anna Masato, Ludovica Iovino, Ilaria Battisti, Giorgio Arrigoni, Derya Shimshek, Elisa Greggio, Marie-Eve Tremblay, Luigi Bubacco, Anna Erlandsson, Laura Civiero

**Affiliations:** 1 Department of Public Health and Caring Sciences, Uppsala University, Uppsala, Sweden; 2 Department of Biology, University of Padova, Padua, Italy; 3 Parkinson and Movement Disorders Unit, Department of Neuroscience, University of Padova, Padua, Italy; 4 PNC, Padua Neuroscience Center, University of Padova, Padua, Italy; 5 IRCCS San Camillo Hospital, Venice, Italy; 6 Department of Biomedical Sciences, University of Padova, Padua, Italy; 7 CRIBI Biotechnology Center, University of Padova, Padua, Italy; 8 Novartis Institutes of BioMedical Research, Basel, Switzerland; 9 Division of Medical Sciences, University of Victoria, Victoria, Canada

**Keywords:** Parkinson’s disease, α-Synuclein, LRRK2, Astrocytes, Glia, Neurodegeneration

## Abstract

Parkinson’s disease (PD) is a neurodegenerative, progressive disease without a cure. To prevent PD onset or at least limit neurodegeneration, a better understanding of the underlying cellular and molecular disease mechanisms is crucial. Mutations in the leucine-rich repeat kinase 2 (*LRRK2*) gene represent one of the most common causes of familial PD. In addition, *LRRK2* variants are risk factors for sporadic PD, making LRRK2 an attractive therapeutic target. Mutations in LRRK2 have been linked to impaired alpha-synuclein (α-syn) degradation in neurons. However, in which way pathogenic LRRK2 affects α-syn clearance by astrocytes, the major glial cell type of the brain, remains unclear. The impact of astrocytes on PD progression has received more attention and recent data indicate that astrocytes play a key role in α-syn-mediated pathology. In the present study, we aimed to compare the capacity of wild-type astrocytes and astrocytes carrying the PD-linked G2019S mutation in *Lrrk2* to ingest and degrade fibrillary α-syn. For this purpose, we used two different astrocyte culture systems that were exposed to sonicated α-syn for 24 h and analyzed directly after the α-syn pulse or 6 days later. To elucidate the impact of LRRK2 on α-syn clearance, we performed various analyses, including complementary imaging, transmission electron microscopy, and proteomic approaches. Our results show that astrocytes carrying the G2019S mutation in *Lrrk2* exhibit a decreased capacity to internalize and degrade fibrillar α-syn via the endo-lysosomal pathway. In addition, we demonstrate that the reduction of α-syn internalization in the Lrrk2 G2019S astrocytes is linked to annexin A2 (AnxA2) loss of function. Together, our findings reveal that astrocytic LRRK2 contributes to the clearance of extracellular α-syn aggregates through an AnxA2-dependent mechanism.

**Supplementary Information:**

The online version contains supplementary material available at 10.1007/s12035-021-02327-8.

## Background

Parkinson’s disease (PD) is the second most common neurodegenerative disease. Yet, there is no cure available and much is unknown regarding the underlying disease mechanisms. PD patients not only suffer from severe motor deficiencies, but also develop non-motor symptoms, including loss of smell, sleep disturbance, depression, and dementia [1]. The main pathological hallmarks of PD are (i) degeneration of dopaminergic neurons in substantia nigra pars compacta and (ii) intracellular inclusions of insoluble alpha-synuclein (α-syn) fibrils [2]. In addition to neuronal inclusions, α-syn deposits appear frequently in astrocytes, at all stages of PD [3–7]. Recent data from our research group suggest that the internalization and accumulation of fibrillar α-syn in astrocytes may play an important role in PD progression and associated chronic neuroinflammation [8, 9]. However, the precise molecular pathways crucial for α-syn clearance by astrocytes currently remain unclear. The aim of the present study was to investigate the role of leucine-rich repeat kinase 2 (LRRK2) in the uptake and degradation of fibrillary α-syn by primary cultured astrocytes.

LRRK2 is a multi-module kinase protein, expressed in various organs and cell types including astrocytes [10–12]. Mutations in LRRK2 are the most frequent cause of familial PD, with seven pathogenic mutations identified (G2019S, I20202T, R1441C/G/H, Y1699C, N1437H) [13]. Because all mutations increase kinase activity in cells [14], LRRK2 is one of the most pursued therapeutic targets in PD. The common G2019S (GS) mutation accounts for ~ 1% of sporadic PD and up to 40% of familial PD in certain populations [15]. LRRK2 exerts pleiotropic effects at the cellular level by regulating multiple steps of vesicle trafficking and cytoskeletal dynamics [14, 16–18]. Accumulating evidence suggests that LRRK2 controls endogenous α-syn clearance via the endo-lysosomal pathway both in vitro and in vivo [19–21]. Moreover, LRRK2 regulates the ingestion of extracellular material by different phagocytic cells. Macrophages and microglia derived from LRRK2 G2019S patients and mice display increased phagocytic capacity, while loss of LRRK2 was shown to induce phagocytic deficits in myeloid cells [22]. In line with these findings, inhibition of LRRK2 kinase activity blocks phagocytosis in microglial cells [23] and normalizes lysosome defects in GBA1-mutant astrocytes [24]. However, LRRK2 has also been shown to impair phagosome maturation in macrophages [25], and to be recruited at later stages of the internalization in macrophages and microglia [26], indicating that a clear understanding of LRRK2’s role in phagocytosis is missing. Of note, a recent report indicates that LRRK2 negatively regulates the clearance of extracellular particles via macropinocytosis [27].

Despite intensive research progress, the exact mechanisms by which LRRK2 mutations lead to neurodegeneration remain largely unclear, especially, when it comes to astrocytes. Being the most abundant glial cell, astrocytes play an important role in maintaining brain homeostasis [28]. Their functions include metabolic support to neurons, modification of synapse signaling, recycling of neurotransmitters, and contribution to the blood-brain barrier required to prevent homeostatic disturbance and inflammation from the periphery. In the PD brain, astrocytes are converted to a reactive, inflammatory state in which they phagocytose aggregated proteins and cell debris, as well as secrete various cytokines and chemokines. Our previous data show that materials, such as dead cell debris and aggregated, pathogenic proteins are internalized and stored in the astrocytes, rather than digested [9, 29–32].

Compelling evidence demonstrates that pathological α-syn can transfer from cell to cell in the PD brain and thereby contribute to disease progression. In cell culture experiments, aggregated α-syn can transfer from neurons to astrocytes and from one astrocyte to another [9, 33]. Hence, regulation of α-syn uptake by astrocytes may indirectly have an impact on PD progression. In the present study, we show that LRRK2 affects α-syn uptake, rather than its degradation, in cultured astrocytes in several ways.

## Methods

### Animals

C57Bl/6J Lrrk2 wild-type (*Lrrk2*^*+/+*^) and knock-out (*Lrrk2*^*−/−*^) mice were respectively provided by Dr. Heather Melrose and Jackson Laboratory (B6.129X1 (FVB)-*Lrrk2* ^*tm1.1Cai*^/J) [34, 35]. Lrrk2 G2019S knock-in mice, backcrossed on a C57Bl/6J background, were used. Lrrk2 G2019S knock-in mice (*Lrrk2*^*GS/GS*^*)* were obtained from Prof. Michele Morari and Novartis Institutes for BioMedical Research, Novartis Pharma AG (Basel, Switzerland) [36]. Housing and handling of mice were done in compliance with national guidelines. All animal procedures were approved by the Ethical Committee of the University of Padova and the Italian Ministry of Health (license 46/2012 and 105/2019).

### Cortical Stem Cell–Derived Astrocytes

Mouse embryonic cortices (gestation days 12–14) were dissected in Hank’s Buffered Salt Solution (HBSS) supplemented with 100 U/ml penicillin, 100 μg/ml streptomycin, and 8 mM HEPES buffer (all Gibco) and dissociated by brief trituration. Blood cells were allowed to sediment for 10 min. The supernatant was transferred and the cells collected by centrifugation (100×*g*, 5 min) followed by careful re-suspension in serum-free proliferation medium containing Dulbecco’s Modified Eagle Medium (DMEM)/F12 with GlutaMAX, 1× B27 supplement, 100 U/ml penicillin, 100 μg/ml streptomycin, 8 mM HEPES buffer (all Gibco) and fortified with 10 ng/ml basic fibroblast growth factor (bFGF, Gibco) and 20 ng/ml epidermal growth factor (EGF, Corning). The embryonic cortical stem cells were allowed to expand as neurospheres in non-treated tissue cultures flasks (at 37 °C, 5% CO_2_) with a passage every 2 to 3 days comprising a thorough dissociation in HBSS and re-suspension in proliferation medium (compositions as described above). For experiments, neurospheres were dissociated in HBSS, taking care to reach a single-cell suspension, and seeded as a monolayer at a density of 2×10^4^ cells/cm^2^ on cover glasses coated with poly-L-ornithine (Sigma-Aldrich) and laminin (Gibco). For the first 24 h, cells were cultured (at 37 °C, 5% CO_2_) in proliferation medium, thereafter in mitogen-free differentiation medium containing DMEM/F12 with GlutaMAX, 1× B27 supplement, 100 U/ml penicillin, 100 μg/ml streptomycin, and 8 mM HEPES buffer (all Gibco), which was fully replaced every 2 to 3 days during the 7-day differentiation period. Being based on embryonic, cortical stem cells, this well-characterized cell culture system solely contains cells of the neural lineage [29, 37–39]. Differentiation led to a mixed population of 75 ± 8% astrocytes, 25 ± 8% neurons, and 6 ± 3% oligodendrocytes, without any microglia or macrophages, as expected from the literature [31, 32, 40]. Independent experiments were carried out using cells obtained from embryos of different pregnant mice.

### Primary Striatal Astrocytes

Mouse primary striatal astrocytes were obtained from postnatal animals between days 1 and 3. Brains were dissected from the skull and placed in a dish, containing cold Dulbecco’s Phosphate Buffered Saline (DPBS, Biowest). Olfactory bulbs and cortices were removed under an optic microscope, and striatum was transferred to a separate dish containing cold DPBS. After the dissection, Basal Medium Eagle (BME, Biowest), supplemented with 10% fetal bovine serum (FBS, Corning), 100 U/ml penicillin, and 100 μg/ml streptomycin, was added to the tissues. Striata were then sifted through a 70-μm cell strainer (Sarstedt), using a syringe plunger. The cell suspension was centrifuged (300×*g*, 15 min), and the pellet was washed two times with 25 ml of supplemented medium. Cells were seeded at a density of 5×10^6^ cells/10 ml medium in cell culture flasks. The culture medium was changed after 7 days and again after additional 3–4 days. When cell confluency reached about 80%, microglia were detached by shaking the flask (800 rpm) for 2 h at room temperature (RT). After shaking, the medium containing microglia was replaced with fresh medium. Cells were maintained in BME supplemented with 10% FBS, 100 U/ml penicillin, and 100 μg/ml streptomycin at 37 °C in controlled 5% CO_2_ atmosphere. Independent experiments were carried out using cells obtained from different pups.

### Immortalized H4 Cells

Human H4 neuroglioma cells were kindly provided by Prof. Patrick A. Lewis (The Royal Veterinary College, London, UK). H4 cells were cultured in DMEM (Biowest) supplemented with 10% FBS (Corning), 100 U/ml penicillin, and 100 μg/ml streptomycin at 37 °C and 5% CO_2_. Cells were plated on 100-mm Ø dishes (Corning) at a density of 5×10^6^ cells/10 ml medium for purification procedure or on 12-mm Ø glass coverslips (Thermo Scientific) at a density of 0.1−10^6^ cells for immunocytochemistry experiments.

### Cell Transfection

Once reached 80% of confluency in 100-mm Ø dishes, H4 cells were transiently transfected with 10 μg of pCHMWS-3xflag-LRRK2 plasmids encoding for human LRRK2 [41] and 5 μg of pEGFP-N3-AnxA2-GFP encoding for human annexin A2 (Addgene, #107196), using linear polyethylenimine (PEI, Polysciences) and following 1:2 DNA to PEI ratio. On 12-mm Ø glass coverslips, H4 cells were transiently transfected with 2 μg of pEGFP-N3-AnxA2-GFP at 60% of confluency. DNA and PEI were diluted in OPTIMEM (Gibco) and incubated for 20 min at RT to allow the formation of DNA/PEI complexes. The mix was added to cells and the experimental procedure was carried out after 48 h. Primary striatal astrocytes were seeded in 24-well plates at the seeding density of 0.025 × 10^6^/well. Cells were transfected with mouse AnxA2 siRNA SMARTPool at a final concentration of 50 nM (Dharmacon) using Lipofectamine 2000 (Thermo Scientific) following 1:3 siRNA to Lipofectamine ratio. After 72 h, the experimental procedure was carried out.

### Alpha-Synuclein

To generate α-syn pre-formed fibrils (PFFs), in-house purified as in [42] or commercial (Anaspec #AS-55555) human α-syn monomers reconstituted at a concentration of 5 mg/ml in sterile DPBS (Gibco) and sterile-filtered (Costar Spin-X centrifuge tube filters (Merck), 0.45 μm) were incubated on a shaker (IKA MS3 Basic or ThermoMixer F1.5 Eppendorf, 1000 rpm) at 37 °C for 7 days. α-syn PFFs were diluted to a stock concentration in sterile PBS and stored at −70 °C. Both unlabeled and Cy3- or SNARF-1 C2-Maleimide-labeled α-syn PFFs were used in this study.

α-syn was labeled using a Cy3 labeling kit (GE Healthcare #PA33000) in accordance with the manufacturer’s instructions. Briefly, 1 mg of α-syn PFFs was mixed with coupling buffer and added to the Cy3 dye. After a 1-h labeling reaction at RT, unbound Cy3 dye was removed by the following wash procedure, which was repeated three times: centrifugation (20,000×*g*, 30 min), removal of supernatant, and re-suspension of the pellet in sterile PBS. Cy3-labeled α-syn PFFs were stored at −70 °C.

α-syn monomer presenting two additional residues at C-terminal position (α-syn Gly-Cys) was prepared as described previously [42, 43] and labeled with SNARF-1 C2-Maleimide dye (Setareh Biotech). SNARF molecule absorbs at 488 nm and changes the emission spectrum at 550 and 630 nm according to the environmental pH. Specifically, the ratio 550/630 nm enhances upon a decrease in pH. By following the procedure described in [44], reduced α-syn Gly-Cys monomers were diluted 1:1 in water and EDTA (1 mM) pH 8 with the addition of 70% ammonium sulfate salt (w/v). Labeling reaction was achieved by adding 5 molar equivalents of SNARF-1 C2-Maleimide dye and incubating at RT overnight. SNARF-1-labeled α-syn was precipitated by centrifugation (20,000×*g*, 10 min, 4 °C), and the exceeding dye was removed through PD-10 column (GE Healthcare). To generate SNARF-1-labeled PFFs, monomers of α-syn and SNARF-labeled α-syn were incubated in a 10:1 ratio. Fluorescence spectra of both free SNARF-1 molecule and SNARF-1-labeled PFFs were analyzed at different pH using a Cary Eclipse fluorescence spectrophotometer (Varian), exciting at 488 nm and collecting spectra within 520–660 nm.

Just before use, labeled and unlabeled α-syn PFFs were diluted to a concentration of 1 mg/ml in sterile PBS and sonicated at a power input between 20 and 30 W for 30 s (pulsing on/off) at 4 °C (Sonics Vibra-Cell sonicator or Covaris S2 Ultrasonicator). Characterization of α-syn PFFs was performed by transmission electron microscopy (TEM) using negative staining. α-syn PFFs were diluted 1:10 in distilled H_2_O and placed on a formvar and carbon-coated 200-mesh copper grid (Ted Pella). The sample was directly stained with 2% uranyl acetate. Dried grids were examined by TEM (FEI Tecnai G2) operated at 80 kV with an ORIUS SC200 CCD camera and Gatan Digital Micrograph software (both Gatan Inc.).

### Alpha-Synuclein Exposure

Cells were exposed to sonicated Cy3-labeled, SNARF-1-labeled, or unlabeled α-syn PFFs at a concentration of 0.5 μM for 24 h. To inhibit Lrrk2 kinase activity, MLi-2 at a concentration of 200 nM (Tocris Bioscience) was applied 90 min before α-syn PFFs treatment and maintained for the entire PFFs exposure. Cells were then rinsed twice with culture medium to remove any excess α-syn PFFs. Subsequently, cells were either processed for immunocytochemistry, western blot, or live imaging assay (24-h time point) or cultured in differentiation medium (DMEM/F12 with GlutaMAX, 1× B27 supplement, 100 U/ml penicillin, 100 μg/ml streptomycin, and 8 mM HEPES buffer (all Gibco)) for 6 additional days (24 h + 6 d time point).

### Immunocytochemistry

Cells were fixed in 4% paraformaldehyde (PFA)/PBS for 20 min at RT, followed by two washes in PBS. Then, cells were permeabilized and blocked in 0.1% Triton X-100/PBS with 5% normal goat serum (NGS) for 30 min at RT or with 5% v/v FBS in PBS for 60 min at RT. Primary antibody incubation was performed using chicken polyclonal glial fibrillary acidic protein (GFAP) (Abcam #ab4674, 1:400), rat monoclonal lysosome–associated protein (Lamp-1) (clone 1D4B) (Abcam #ab25245, 1:100), rabbit polyclonal lysosome–associated membrane protein (Lamp2A) (Abcam #ab18528, 1:200), purified mouse anti-α-synuclein (BD Laboratories #610787, 1:400), rabbit polyclonal annexin II (GeneTex International #GTX101902, 1:200). Secondary antibody incubation was carried out for 30 min at 37 °C or for 1 h at RT using anti-chicken Alexa Fluor 647 (Invitrogen #A21449), anti-rabbit Alexa Flour 488 (Invitrogen #A11034), and anti-mouse Alexa Fluor 568 (Invitrogen #A11004) fluorophores. Secondary antibodies were diluted 1:200 in 0.1% Triton X-100/PBS with 0.5% NGS or with 5% v/v FBS in PBS. Both primary and secondary antibody incubations were followed by three washes in PBS for 5 min. In some experiments, cells were stained using Phalloidin-iFluor 647 Reagent (Abcam #ab176759). Cover glasses were mounted on a microscope slide (Thermo Scientific) using EverBrite Hardset mounting medium (Biotium) or Mowiol (Calbiochem) supplied with DAPI and, in some experiments, in combination with VECTASHIELD HardSet antifade mounting medium with TRITC-phalloidin (Vector Laboratories).

### Transmission Electron Microscopy and Image Analysis

Primary striatal astrocytes were seeded onto 24-well plates (10×10^4^ cells) and fixed at 80% confluency. The medium was removed, and fixative buffer (glutaraldehyde 2.5% in 0.1 M sodium cacodylate buffer) was added to the cells for 1 h at 4 °C. Then, the samples were post-fixed using 1% osmium tetroxide plus potassium ferrocyanide 1% in 0.1 M sodium cacodylate buffer for 1 h at 4 °C. After three washes with water, samples were dehydrated in a graded ethanol series and embedded in epoxy resin (Epoxy Embedding Medium kit, Sigma-Aldrich). Ultrathin sections (60–70 nm) were obtained with an Ultrotome V (LKB) ultramicrotome, counterstained with uranyl acetate and lead citrate, and viewed with a Tecnai G2 (FEI) transmission electron microscope operating at 100 kV. Images were captured, using a Veleta (Olympus Soft Imaging System) magnification ×9800 digital camera. Electron microscopy images were analyzed using ImageJ and blind to the experimental conditions. We identified lysosomal-like structures using the following ultrastructural features: 0.05–0.5 μm in diameter and granular, electron-dense appearance in electron micrographs [45]. Lysosomal-like structures were counted, and their areas were determined using the oval selection tool of the Region of Interest (ROI) Manager tool (ImageJ). Lysosomal-like structure numbers were normalized to the field area. The number of independent cell cultures used was as follows: *Lrrk2*^+/+^, *n* = 4; *Lrrk2*^−/−^, *n* = 4; and *Lrrk2*^GS/GS^, *n* = 4. For each cell culture, fifty independent fields were analyzed for quantification.

### Fluorescence Microscopy and Image Analysis

For cortical stem cell–derived astrocyte studies, images were taken with a Zeiss Axio Observer Z1 fluorescence microscope equipped with a ×40/0.93 NA plan-apochromat objective and a ×63/1.40 oil DIC plan-apochromat objective. Images were acquired at a 16-bit intensity resolution over 2048 × 2048 pixels. The number of independent cell cultures used was as follows: *Lrrk2*^+/+^, *n* = 7; *Lrrk2*^−/−^, *n* = 6; and *Lrrk2*^GS/GS^, *n* = 5 at the 24-h time point and *Lrrk2*^+/+^, *n* = 6; *Lrrk2*^−/−^, *n* = 6; and *Lrrk2*^GS/GS^, *n* = 5 at 24 h + 6 d. For each culture, ten independent fields per cover glass were evaluated and reported. Cy3 α-syn inclusions were analyzed using ImageJ. An ImageJ macro was developed that included the following steps: set scale, convert to 8-bit, subtract background, set threshold (the same threshold was used for both time points), set measurements, and analyze particles. In each image, Cy3 α-syn deposits were assessed by measuring the total area taken up by the Cy3 signal, by the number of particles counted as well as by the sum of the integrated densities (area × mean intensity of each Cy3 α-syn deposit). Analysis results were normalized to the number of living cells (identified by DAPI staining). Since various sized Cy3 α-syn inclusions were observed, the ImageJ macro was adjustable to quantify different types of inclusions separately. Judging by the area measurements of the various particles, the cut-off limit for the group of small Cy3 α-syn particles was set at ≤25 μm^2^. Quantifications for larger (>25 μm^2^) Cy3 α-syn particles were obtained by subtracting the area/particle count/integrated density information of the small Cy3 α-syn particles from numbers acquired when quantifying all Cy3 α-syn particles.

For primary striatal astrocytes – live imaging studies, images were acquired at 8-bit intensity resolution over 1024 × 1024 pixel, through Leica SP5 confocal microscope, using a HC PL FLUOTAR ×20/0.50 dry objective. The number of independent cell cultures used was as follows: *Lrrk2*^+/+^, *n* = 4; *Lrrk2*^−/−^, *n* = 4; and *Lrrk2*^GS/GS^, *n* = 4. Pictures were acquired at the two relevant ranges of the emission spectrum (channel1: 530–550 nm and channel2: 610–630 nm). For each culture, six to eight independent fields were evaluated and reported. SNARF-1-positive α-syn inclusions were analyzed using ImageJ. SNARF-1-labeled α-syn PFFs were assessed using an ImageJ macro including the following steps: set scale, convert to 8-bit, subtract background, set threshold, set measurements, and analyze particles. The ratio of the single-particle integrated density (area × mean intensity) between channel1 and channel2 and the number of particles per ROI were measured. ROIs were first identified in channel1 and then transferred to channel2.

For primary striatal astrocytes – post-fixation imaging, images were acquired at 8-bit intensity resolution over 1024 × 1024 pixels, through Leica SP5 confocal microscope using a HC PL FLUOTAR ×40/0.70 dry objective. Lamp2A-positive puncta were counted using Analyze Particles plug-in in ImageJ. Fluorescent puncta were assessed by area and particle count. The number of independent cell cultures used for the evaluation of Lamp2A-positive puncta in *Lrrk2*^+/+^ versus *Lrrk2*^−/−^ versus *Lrrk2*^GS/GS^ astrocytes was as follows: *Lrrk2*^+/+^, *n* = 3; *Lrrk2*^−/−^, *n* = 3; and *Lrrk2*^GS/GS^, *n* = 3. For each culture, eight independent fields per experiment were evaluated and reported. Proximity analysis for AnxA2 and unlabeled α-syn PFFs dots was performed using ComDet plug-in in ImageJ (https://imagej.net/Spots_colocalization_(ComDet)), using the following parameters: max co-localization distance (0.9 pixels) and particles dimension (AnxA2: 3 pixels and α-syn: 4 pixels). The number of AnxA2 dots and the number of α-syn puncta were assessed by “puncta count” output and the proximity between AnxA2 dots and engulfed α-syn as “co-localization” output. All quantifications were normalized to the number of living cells identified by nuclei staining. The number of independent cell cultures used in AnxA2 downregulation experiments was *Lrrk2*^+/+^ *n* = 3. For each culture, four independent fields per experiment were evaluated and reported. The number of independent cell cultures used for the evaluation of AnxA2 function in *Lrrk2*^+/+^ versus *Lrrk2*^GS/GS^ astrocytes was as follows: *Lrrk2*^+/+^, *n* = 3; *Lrrk2*^GS/GS^, *n* = 3. For each culture, eight independent fields per experiment were evaluated and reported.

### Protein Purification

3xFlag-LRRK2 purification was performed as described in [41]. Briefly, transfected H4 cells were solubilized in an appropriate volume of radioimmunoprecipitation assay buffer, RIPA buffer (20 mM Tris-HCl pH 7.5, 150 mM NaCl, 1 mM EDTA, 2.5 mM sodium pyrophosphate (Na_4_P_2_O_7_), 1 mM β-glycerophosphate (C_3_H_7_Na_2_O_6_P), 1 mM sodium orthovanadate (Na_3_VO_4_)) containing 1% protease inhibitor cocktail (Sigma-Aldrich). Lysates were centrifugated for 30 min at 14,000×*g*. Afterwards, lysates containing 3xFlag-tagged protein were incubated with anti-Flag M2 agarose beads for 2 h at 4 °C on a rotator. After extensive washing, proteins were eluted with 150 ng/ml of 3xFlag peptide by shaking for 30–40 min at 4 °C. Proteins were then resolved by SDS-PAGE and stained for 2 h with Colloidal Coomassie Brilliant Blue (0.1% w/v Brilliant Blue G-250, 25% v/v methanol, 5% v/v acetic acid and milli-Q water) for 1 h. Then, destained with a colloidal destaining (7.5% v/v acetic acid, 5% v/v methanol and milli-Q water). Finally, gel band was excised and assessed by mass spec (see below).

### Western Blot Analysis

Cells were lysed in an appropriate volume of RIPA buffer (20 mM Tris-HCl pH 7.5, 150 mM NaCl, 1 mM EDTA, 0.5 mM sodium pyrophosphate (Na_4_P_2_O_7_), 1 mM β-glycerophosphate (C_3_H_7_Na_2_O_6_P), 1 mM sodium orthovanadate (Na_3_VO_4_)) containing 1% protease inhibitor cocktail (Sigma-Aldrich). Protein concentration was determined through the Pierce BCA Protein Assay Kit following the manufacturer’s instructions (Thermo Scientific) and 25 μg of each sample was prepared for SDS-PAGE with the addition of sample buffer 4×. Electrophoresis was performed using ExpressPlus PAGE precast gels 4–20% (GeneScript), according to the manufacturer’s instructions. After electrophoresis, protein samples were transferred to PVDF membranes (Bio-Rad) through a Trans-Blot Turbo^TM^ Transfer System (Bio-Rad) in semi-dry conditions, with the 1× transfer buffer (Bio-Rad) at 25 V for 20 min. Proteins were identified by the appropriate primary antibodies against LRRK2 (Abcam #ab133474, 1:300), β-actin (Sigma-Aldrich #A1978, 1:10000), annexin II (GeneTex International #GTX101902, 1:1000), alpha-synuclein (Abcam #ab138501, 1:10000), Flag M2-peroxidase (Sigma-Aldrich #A8592, 1:10000); and then incubated for 1 h at RT with appropriate horseradish peroxidase (HRP)–conjugated secondary antibodies (Invitrogen). The visualization of the signal was conducted using Immobilon Forte Western HRP substrate (Millipore) and the VWR Imager Chemi Premium. Images were acquired in tiff format and processed by the ImageJ software to quantify the total intensity of each single band.

### Mass Spectrometry Analysis

Gel slices were cut into small pieces and subjected to reduction with dithiothreitol (DTT 10 mM in 50 mM NH_4_HCO_3_, for 1 h at 56 °C), alkylation with iodoacetamide (55 mM in 50 mM NH_4_HCO_3_, for 45 min at RT and in the dark), and finally in-gel digestion with sequencing grade modified trypsin (12.5 ng/μL in 50 mM NH_4_HCO_3_, Promega) as reported in [46]. Samples were analyzed using a LTQ Orbitrap XL mass spectrometer (Thermo Fisher Scientific) coupled to a HPLC UltiMate 3000 (Dionex – Thermo Fisher Scientific) through a nanospray (NSI). Peptides were separated at a flow rate of 250 nL/min using an 11-cm-long capillary column (PicoFrit, 75-μm ID, 15-μm tip, New Objective) packed in house with C18 material (Aeris Peptide 3.6 μm XB C18; Phenomenex). A linear gradient of acetonitrile/0.1% formic acid from 3 to 40% was used for peptide separation and the instrument operated in a data dependent acquisition mode with a Top4 method (one full MS scan at 60,000 resolution in the Orbitrap, followed by the acquisition in the linear ion trap of the MS/MS spectra of the four most intense ions). Raw data files were analyzed using Proteome Discoverer 1.4 (Thermo Fisher Scientific) connected to a Mascot local server (version 2.2.4, Matrix Science) and searched against the human section of the UniProt database (version July 2018, 95057 entries) using the following parameters: trypsin was selected as digesting enzyme with up to one missed cleavage allowed, precursor and fragment tolerance was set to 10 ppm and 0.6 Da respectively, carbamidomethylation of cysteine residues was set as a fixed modification and methionine oxidation as a variable modification. The precursor area ion detector node of Proteome Discoverer was used to integrate the area of precursor ions. A search against a randomized database and the algorithm Percolator were used to assess the false discovery rate (FDR), and data were filtered to keep into account only proteins identified with at least two unique peptides and a FDR ≤ 0.01 both at peptide and protein levels. Proteins were grouped into protein families according to the principle of maximum parsimony.

### Measurement of Lysosomal pH

Cells were plated in lumox 96 multiwell (40×10^3^ cells) (Sarstedt) and treated with α-syn PFFs (0.5 μM, 24 h) once reached 95% of confluency with or without the autophagy inhibitor bafilomycin (50 nM, 1 h). Cells were then rinsed once and treated with the ratiometric dye, 2-(4-pyridyl)-5-((4-(2-dimethylaminoethylaminocarbamoyl) methoxy) phenyl) oxazole (RatioWorks PDMPO, AAT Bioquest) at a final concentration of 2 μM in OPTIMEM for 5 min. After the incubation, cells were rinsed three times in OPTIMEM and fluorescence was measured at 37 °C using a multi-plate reader (EnVision, Perkin Helmer). Specifically, the emitted fluorescence at 535 nm was acquired upon excitation at 340 nm and 380 nm. The ratio of the light excited at two wavelengths (340/380 nm) is proportional to lysosomal pH. For each sample, a replicate of two wells was used to determine the ratio.

### Neutral Red Staining

Cells were plated in 24-well plates (10×10^4^ cells) and treated with α-syn PFFs (0.5 μM, 24 h) once reached 80% of confluency with or without bafilomycin (50 nM, 1 h); at the end of the treatment, the cell culture medium was removed and OPTIMEM with a solution of 3-amino-7-dimethylamino-2-methyl-phenazine hydrochloride (neutral red, Sigma-Aldrich) with a final concentration of 40 ng/ml was added to the cells for 3–4 h. Cells were washed twice with DPBS and dissolved in a destaining solution composed of 50% ethanol, 49% deionized water, and 1% glacial acetic acid, and the absorbance was recorded by the use of a multiwell plate reader at the wavelength of 540 nm (Victor, Perkin Helmer). For each sample, treated and untreated, a replicate of two wells was used to determine protein concentration (BCA assay). Data were expressed as absorbance at 540 nm normalized to the absorbance recorded for the BCA assay, and the final results in the graph were expressed as neutral red staining absorbance in comparison with untreated controls.

### Statistical Analysis

Experiments were performed using cell cultures obtained either from embryos of at least three different pregnant mice or from at least three different groups of pups. H4 cells were used at the same in vitro passage number for the two independent experiments. Results are expressed as mean ± standard error of the mean (SEM) or median with interquartile range; depending on whether data followed a Gaussian distribution or not. Gaussian distribution was assessed by D’Agostino-Pearson omnibus and Shapiro-Wilk normality tests. For Gaussian distribution, the statistical analysis between two groups was performed by unpaired Student’s *t* test. Data including more than two groups were analyzed by one-way ANOVA (Gaussian distribution) or Kruskal-Wallis test (non-Gaussian distribution) respectively followed by Tukey’s multiple comparisons test or Dunn’s multiple comparisons test. Levels of significance were defined as *p* ≤ 0.05, *p* ≤ 0.01, *p* ≤ 0.001. Statistical analysis was performed in Prism 6 (GraphPad).

## Results

### G2019S Lrrk2 Astrocytes Display Impaired Exogenous α-syn Handling

**Fig. 1:**
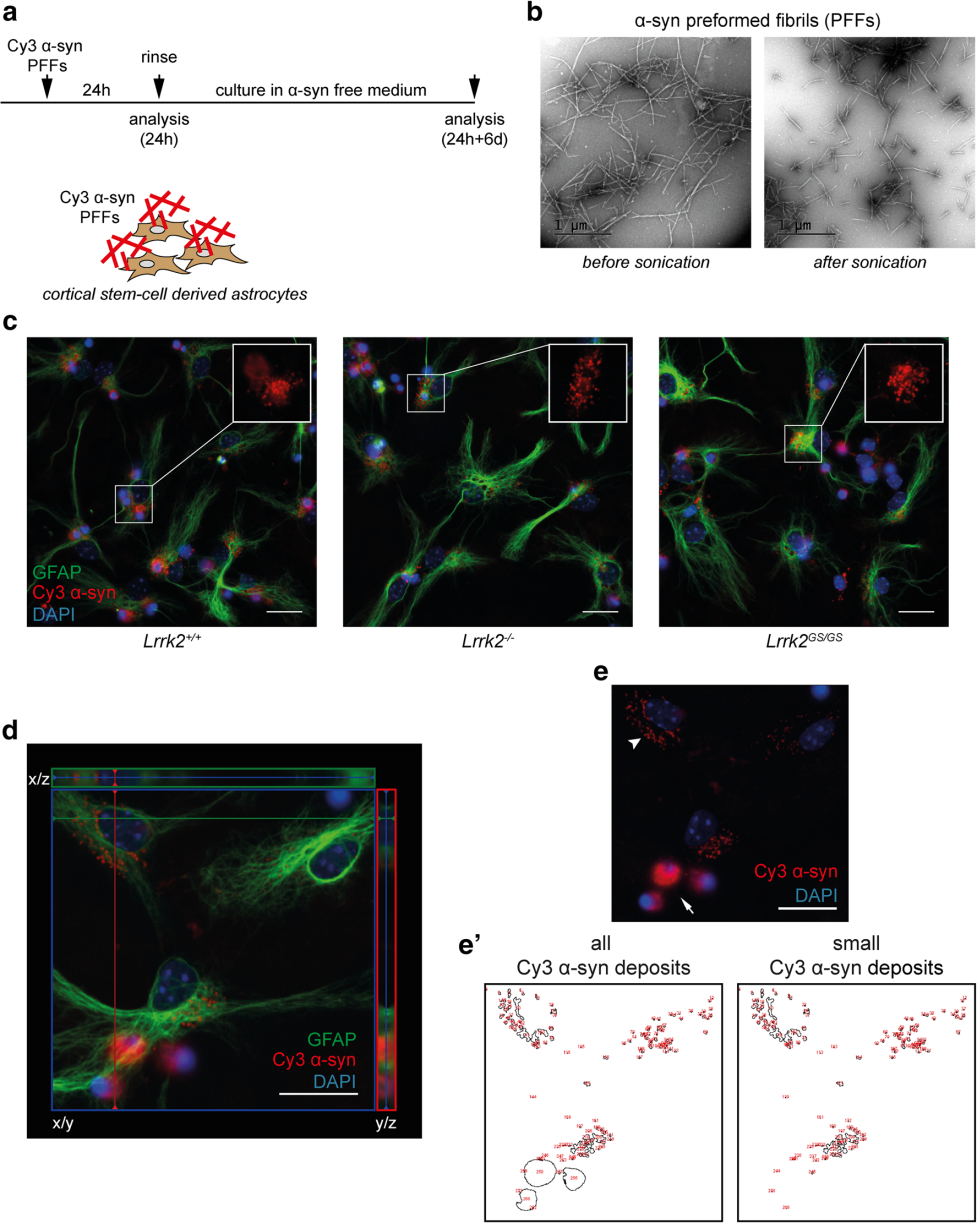
Accumulation of aggregated α-syn in *Lrrk2* astrocytes. **A** Schematic outline of the experimental setup. Cells were exposed to 0.5 μM sonicated Cy3 α-syn PFFs for 24 h. **B** TEM images of α-syn PFFs pre and post sonication. Scale bars 1 μm. **C** Representative fluorescence microscopy images of *Lrrk1*^*+/+*^, *Lrrk2*^*−/−*^, and *Lrrk2*^*GS/GS*^ astrocytes (GFAP, green) at 24 h; cell nuclei stained with DAPI (blue) and α-syn labeled with Cy3 (red). Insets show a close-up of Cy3 α-syn inclusions. **D** Orthogonal projections of z-stack images taken with a fluorescence microscope: main view (x/y), top (x/z), and right (y/z). Projections were made along the lines depicted in the main image. Astrocytes (GFAP, green), Cy3 labeled α-syn (red), DAPI (blue). **E** Fluorescence microscopy image showing the differently sized Cy3 α-syn inclusions observed: small dot-like inclusions (arrow head) and larger, cottony deposits (arrow). **E’** Displays of the particle count obtained from the ImageJ analysis when including either all Cy3 α-syn deposits or only the small Cy3 α-syn inclusions. Scale bars = 20 μm (C, D, and E)

To study how LRRK2 affects exogenous fibrillar α-syn clearance in astrocytes in total absence of microglia, we examined the uptake and degradation capacities of cortical stem cell–derived astrocytes isolated from *Lrrk2*^*−/−*^ and *Lrrk2*^*GS/GS*^ mice. As depicted in Fig. 1A, astrocytes were exposed to 0.5 μM α-syn PFFs for 24 h, thoroughly rinsed and then processed for analysis or incubated for additional 6 days in α-syn-free medium. TEM analysis verified the successful generation of PFFs and confirmed efficient sonication of α-syn PFFs used for the experiments (Fig. 1B).

Comparable to our previous findings [8, 9, 32, 47], astrocytes engulfed and accumulated large amounts of α-syn (Fig. 1C). All intracellular Cy3 α-syn deposits localized around the nucleus of astrocytes. As reported by us before [32, 47], we observed different types of Cy3 α-syn inclusions: small dot-like inclusions and larger, cottony deposits (Fig. 1C). The larger, more diffuse Cy3 α-syn inclusions were also found in close proximity to pyknotic cell nuclei (ingested by the astrocytes during the differentiation period and characterized by a condensed DAPI staining) (Fig. 1C) [30, 32]. Moreover, immunocytochemistry revealed that ingested α-syn was deposited in vesicles expressing the endo-lysosomal marker Lamp-1 (Supplementary Figure 1) [8].

To study the different types of Cy3 α-syn inclusions more closely, z-stack imaging was performed. All Cy3 α-syn deposits were surrounded by or were observed in close proximity to GFAP staining (Fig. 1D). GFAP is an intermediate filament protein that constitutes part of the cytoskeleton of astrocytes but does not encompass the whole cell (Supplementary Figure 2) [48]. GFAP staining thus underestimates the perimeter of astrocytes. Both small dot-like inclusions (Fig. 1E, arrow head) and larger, cottony deposits (Fig. 1E, arrow) were observed, and the ImageJ analysis macro used to quantify the amount of Cy3 α-syn inclusions was adaptable to analyze differently sized Cy3 α-syn deposits separately (Fig. 1E’).

**Fig. 2:**
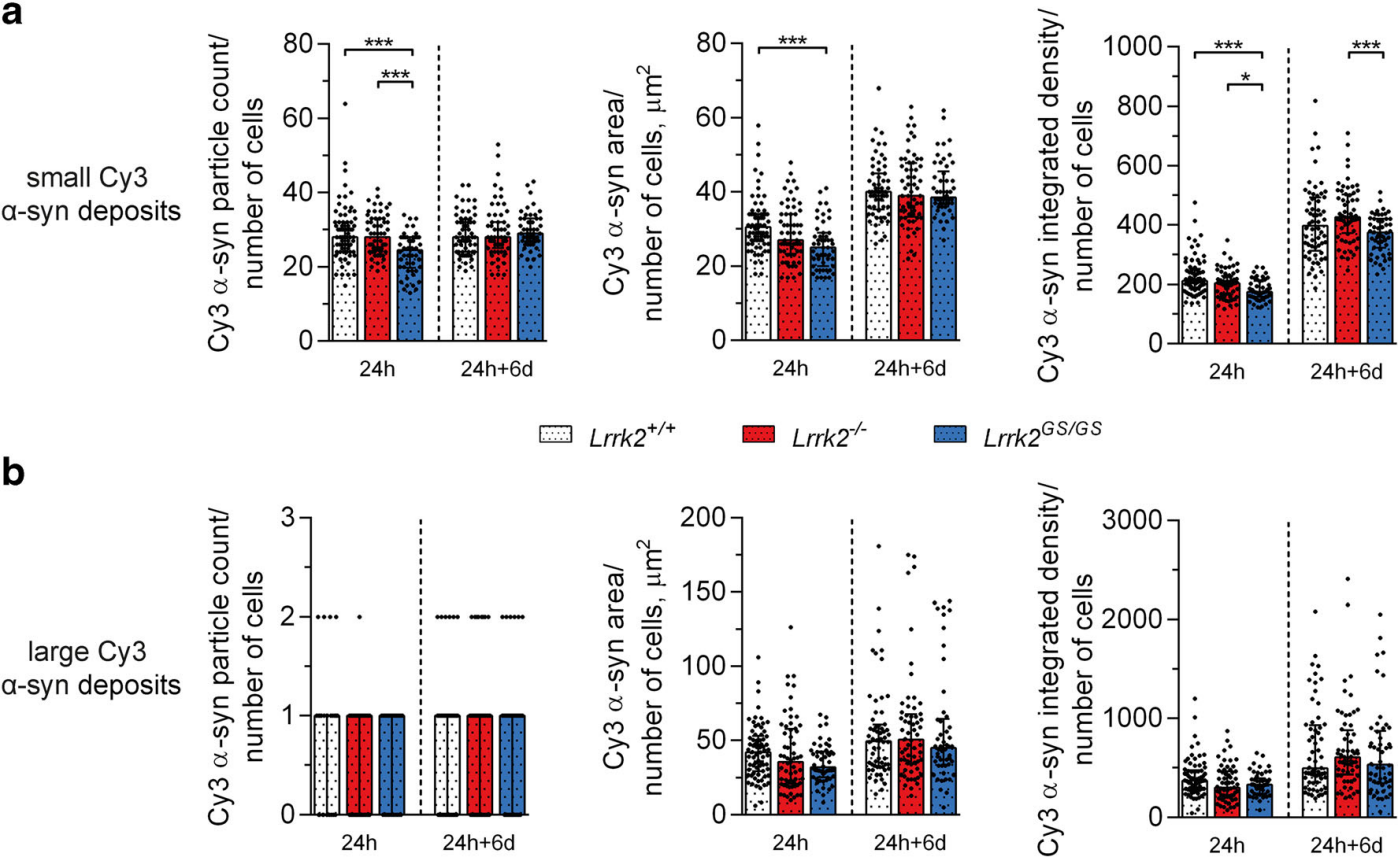
Analysis of the differently sized α-syn inclusions in *Lrrk2* astrocytes. **A**, **B** Analysis of small and large Cy3 α-syn inclusions, respectively. Quantifications of Cy3 α-syn particle count, total area, and integrated density were performed using ImageJ. For both time points, ten images per independent cell culture (24 h: *Lrrk1*^+/+^, *n* = 7; *Lrrk2*^−/−^, *n* = 6; and *Lrrk2*^GS/GS^, *n* = 5; 24 h + 6 d: *Lrrk1*^+/+^, *n* = 6; *Lrrk2*^−/−^, *n* = 6; and *Lrrk2*^GS/GS^, *n* = 5) were analyzed and reported. For each time point, the statistical analysis was performed with the Kruskal-Wallis test followed by Dunn’s multiple comparisons test, since the data did not follow Gaussian distribution for all groups. *p ≤ 0.05, **p ≤ 0.01, ***p ≤ 0.001

The quantification of all Cy3 α-syn particles together revealed that only *Lrrk2*^*GS/GS*^ astrocytes displayed a lower amount of α-syn inclusions (Supplementary Figure 3), a difference that predominantly resulted from the group of small α-syn deposits, as shown by quantifying the differently sized inclusions separately (Fig. 2A and B). Examining the small Cy3 α-syn deposits at the 24 h time point, *Lrrk2*^*GS/GS*^ cells displayed lower values than *Lrrk2*^*+/+*^ for all parameters analyzed (Fig. 2A; *Lrrk2*^*GS/GS*^ vs *Lrrk2*^*+/+*^, *p* ≤ 0.001; Kruskal-Wallis test followed by Dunn’s multiple comparisons test). *Lrrk2*^*GS/GS*^ also displayed lower Cy3 α-syn amounts compared to *Lrrk2*^−/−^ in terms of particle count (*p* ≤ 0.001) and integrated density (*p* ≤ 0.05), but not for the area measurement (Fig. 2A; Kruskal-Wallis test followed by Dunn’s multiple comparisons test). On the other hand, the analysis of larger Cy3 α-syn inclusions did not reveal any statistically significant differences between the LRRK2 genotypes (Fig. 2B, Cy3 α-syn particle count: *p* = 0.7268, Cy3 α-syn area: *p* = 0.0775, Cy3 α-syn integrated density: *p* = 0.0605; Kruskal-Wallis test followed by Dunn’s multiple comparisons test).

At the 24 h + 6 d time point the only statistically significant difference observed for the small Cy3 α-syn particles was the integrated density measurement (Fig. 2A, *Lrrk2*^*GS/GS*^ vs *Lrrk2*^−/−^ *p* ≤ 0.001; Kruskal-Wallis test followed by Dunn’s multiple comparisons test). For the large Cy3 α-syn inclusions, no statistically significant difference between the LRRK2 genotypes was observed for all parameters analyzed at 24 h + 6 d (Fig. 2B, Cy3 α-syn particle count: *p* = 0.7689, Cy3 α-syn area: *p* = 0.8065, Cy3 α-syn integrated density: *p* = 0.5824; Kruskal-Wallis test followed by Dunn’s multiple comparisons test).

Insights on the degradation capacity of astrocytes were gained by observing changes between the two time points (24 h vs 24 h + 6 d). Overall, we confirmed that astrocytes store rather than degrade the aggregated α-syn (Fig. 2). Area and integrated density measurements showed a 1.5- to 2-fold increase for both small and large Cy3 α-syn deposits, while the number of particles counted did not change (apart for *Lrrk2*^*GS/GS*^, which showed a 1.2-fold change increase of small Cy3 α-syn deposits). These results might be explained by the fact that aggregates are brought closer together over the 6-day period. Following ingestion, the α-syn aggregates are relocated inside the cell to end up in “storage dumps” around the nucleus, a phenomenon that we have previously documented using different astrocytic culture systems after astrocytic engulfment of α-syn or amyloid-beta aggregates [9, 31].

Thus, our data indicate that the amount of α-syn present inside *Lrrk2*^*GS/GS*^ astrocytes is reduced compared to wild-type or *Lrrk2*^−/−^ astrocytes. The effect is normalized over time, suggesting that the pathogenic mutation influences the uptake rather than the degradation of α-syn.

### G2019S Striatal Astrocytes Show Reduced Engulfed α-syn

Given the high expression of LRRK2 in the striatum [10, 49, 50], we isolated striatal astrocytes from *Lrrk2*^*GS/GS*^, *Lrrk2*^−/−^, and wild-type mice to further confirm our data. The culture purity was assessed by immunofluorescence against GFAP and Iba1, which are astrocytic and microglial markers, respectively (Supplementary Figure 4A–B). The characterization revealed that 94 ± 2% of cells are identified as astrocytes and 7 ± 2% of cells as microglia (Supplementary Figure 4A–B). Similar to cortical stem cell–derived astrocytes, striatal astrocytes are able to ingest and direct sonicated α-syn PFFs to endo-lysosomal organelles positive for Lamp2A marker (Supplementary Figure 4D).

**Fig. 3:**
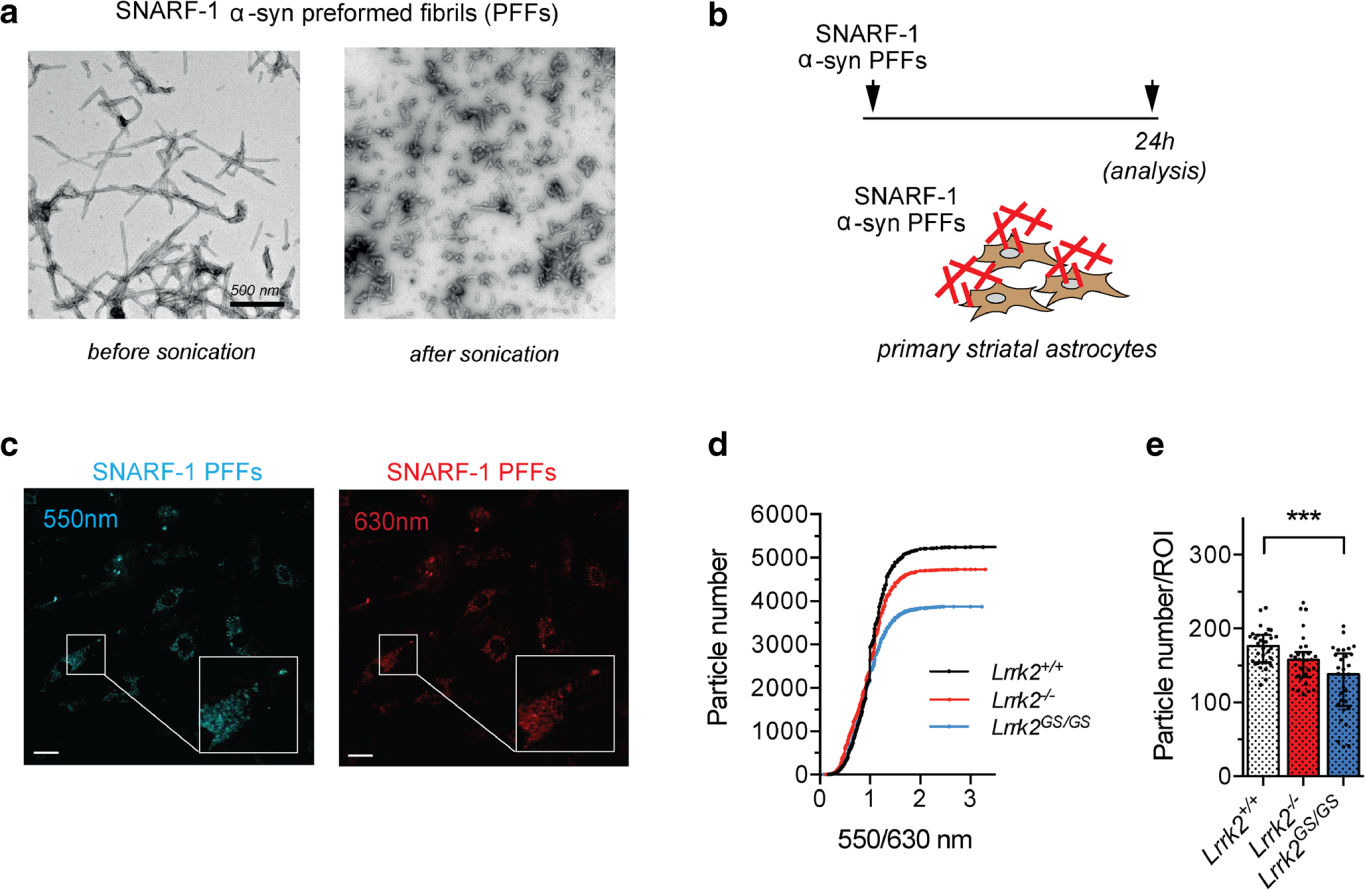
Internalization of aggregated α-syn in striatal astrocytes. **A** TEM images of SNARF-1 α-syn PFFs pre and post sonication. **B** Schematic outline of the experimental setup. Cells were exposed to 0.5 μM sonicated SNARF-1 α-syn PFFs for 24 h and imaged using live confocal laser scanning microscopy. **C** Representative images of primary *Lrrk2*^*+/+*^ striatal astrocytes treated with SNARF-1 α-syn PFFs were acquired at range of 530–550 and 610–630 nm. Scale bar 50 μm. **D**, **E** Eight images per cell culture were analyzed (*n* = 4 independent cultures). For each image, ROIs were traced and quantification of α-syn single-particle 550/630 ratio (IntDen) and number were performed using ImageJ. The cumulative distribution of the single-particle ratio was graphed for each genotype in **D** and particle number per ROI in **E**. Statistical analysis in **E** was performed using Kruskal-Wallis test followed by Dunn’s multiple comparisons test. *p ≤ 0.05, **p ≤ 0.01, ***p ≤ 0.001

To quantify Lrrk2-mediated effects of α-syn PFF clearance in this cellular system, we conjugated monomeric α-syn with a pH-sensitive dye named SNARF-1 C2-Maleimide (SNARF-1 from now on) followed by fibrillization and sonication. As shown by TEM analysis, SNARF conjugation does not affect α-syn PFF aggregation process (Fig. 3A). Relevant to the experimental setup, we ruled out that the chemico-physical properties of the dye are changed upon conjugation and fibrillation process (Supplementary Figure 5). Indeed, the inflection point of free and conjugated SNARF curves are 7.826 (*R* = 0.9956) and 7.606 (*R* = 0.9918), respectively (Supplementary Figure 5). SNARF is particularly suitable to sense pH that ranges between 6 and 9 (Supplementary Figure 5) [51, 52] allowing to monitor α-syn PFFs trafficking through the endo-lysosomal pathway. We treated cells with 0.5 μM SNARF-labeled- α-syn PFFs and captured images after 24 h using live cell confocal microscopy at the two relevant emission ranges (Fig. 3C). Quantification shows that *Lrrk2*^*GS/GS*^, *Lrrk2*^*−/−*^ and wild-type striatal astrocytes display a similar cumulative distribution 550/630 ratio at the single-particle level (Fig. 3D). Therefore, in all the three genotypes, SNARF-labeled α-syn PFFs are distributed in overlapping organelle environments, excluding the possibility that LRRK2 interferes with the flux of extracellular α-syn once internalized as well as the global pH of the organelles. However, similar to what we observed in cortical astrocytes, the total particle number identified in the *Lrrk2*^*GS/GS*^ astrocytes (Fig. 3D) and the number of particles per ROI are significantly reduced (Fig. 3E; *Lrrk2*^*−/−*^ vs *Lrrk2*^*GS/GS*^ PFFs, *p* < 0.001, using Kruskal-Wallis test followed by Dunn’s multiple comparisons test).

Taken together, these results further support that the G2019S pathological mutation in *Lrrk2* modifies the ability of striatal astrocytes to intracellularly accumulate exogenous α-syn.

### Lrrk2 Impacts Endo-lysosomal Capacity in Striatal Astrocytes

**Fig. 4:**
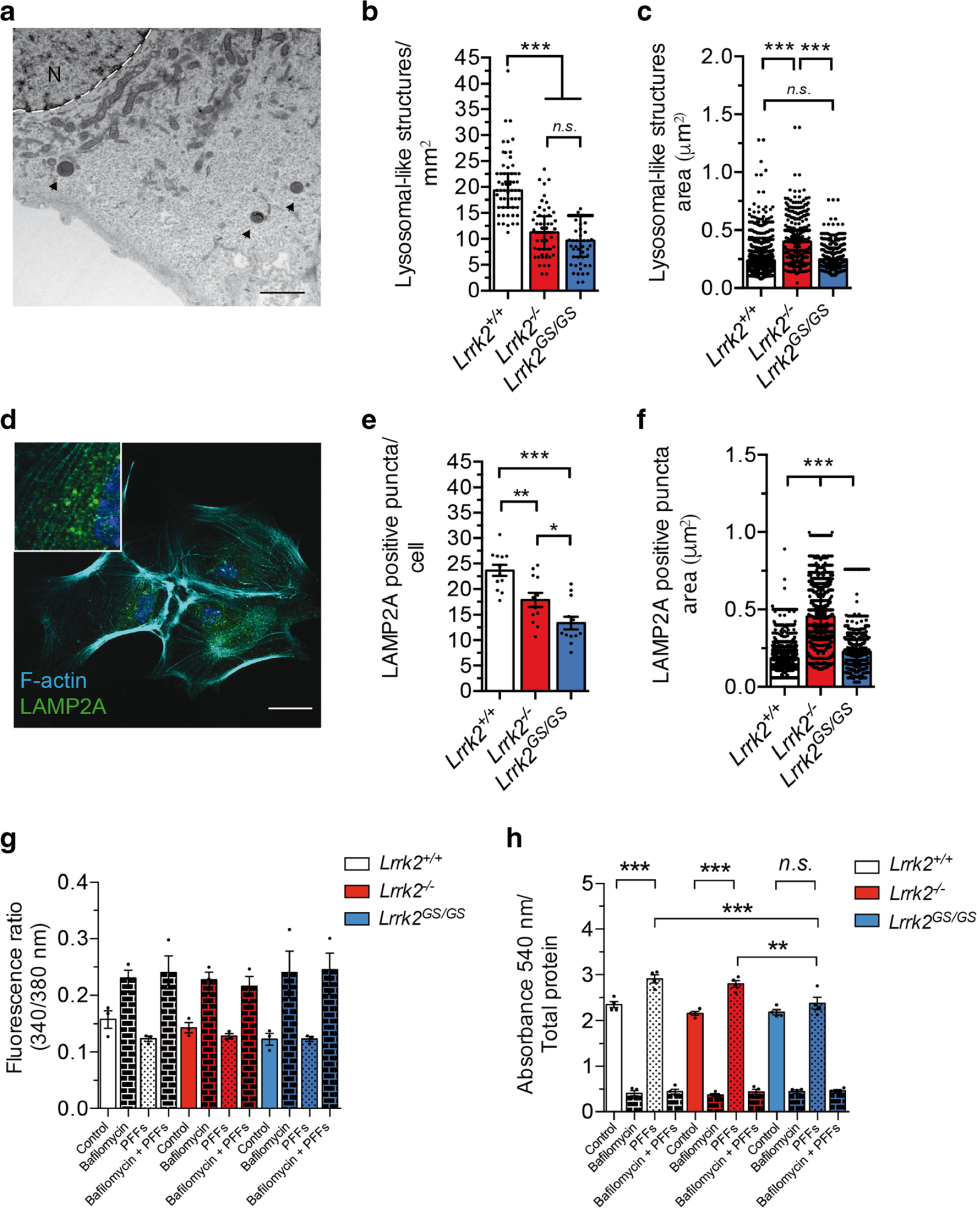
Description of the endo-lysosomal pathway in Lrrk2 striatal astrocytes. **A** Representative TEM image of primary striatal astrocyte section containing electron-dense lysosomal-like structures (arrows). Scale bar 2 μm. **B**, **C** Forty TEM images were acquired (*n* = 4 per genotype). Each cell was imaged by covering the entire cytoplasm and lysosomal-like structure number and area were measured using ImageJ. **D** Representative image of the staining using Lamp2A (green) as a marker for the endo-lysosomal pathway, DAPI (blue) for the nuclei, and F-actin (cyano) to define cells. Scale bar 20 μm. Inset shows a close-up of Lamp2A-positive structures. Quantifications of Lamp2A-positive structure number and area were analyzed using ImageJ. **E**, **F** Four images per cell culture were analyzed (*n* = 3 per genotype). **G** Measurement of lysosomal pH was done in primary striatal astrocytes from the three genotypes upon unlabeled α-syn PPF treatment (*n* = 3 per genotype). Bafilomycin has been applied as negative control. Fluorescence ratio of light acquired at 535 nm upon excitation at 340 and 380 nm is provided. **H** Neutral red assay was performed in primary striatal astrocytes from the three genotypes upon unlabeled α-syn PPF treatment (*n* = 4 per genotype). Bafilomycin has been applied as negative control. Absorbance at 540 nm measured upon cell lysates was normalized by total protein content. Statistical analysis in **B**, **C**, and **F** was made by Kruskal-Wallis test followed by Dunn’s multiple comparisons test. Statistical analysis in **E**, **G,** and **H** was performed with one-way ANOVA followed by Tukey’s multiple comparisons test. *p ≤ 0.05, **p ≤ 0.01, ***p ≤ 0.001

Since the pathogenic mutation in *Lrrk2*^*GS/GS*^ astrocytes impairs their ability to store aggregated α-syn, we next sought to examine the functional aspects of the endo-lysosomal pathway. First, we investigated whether Lrrk2 has an impact on the number and dimension of endo-lysosomal structures in mouse striatal astrocytes. Astrocytes from *Lrrk2*^*+/+*^, *Lrrk2*^*−/−*^, and *Lrrk2*^*GS/GS*^ mice were imaged by TEM and the number and area of lysosomal-like structures were measured (Fig. 4A–C). Quantification showed that both *Lrrk2* ablation and the G2019S pathological mutation caused a significant decrease in the number of lysosomal-like structures in astrocytic cells (Fig. 4A–B; *Lrrk2*^*+/+*^ vs *Lrrk2*^*−/−*^, *p* < 0.001; *Lrrk2*^*+/+*^ vs *Lrrk2*^*GS/GS*^, *p* < 0.001; *Lrrk2*^*GS/GS*^ vs *Lrrk2*^*−/−*^, *p* > 0.05; Kruskal-Wallis test followed by Dunn’s multiple comparisons test) and in the intact striatum (Supplementary Figure 7A–B; *Lrrk2*^*+/+*^ vs *Lrrk2*^*−/−*^, *p* < 0.05; unpaired *t* test). However, the absence of Lrrk2 showed enlarged structures, compared to *Lrrk2*^*+/+*^ as well as *Lrrk2*^*GS/GS*^ astrocytes (Fig. 4A–C; *Lrrk2*^*+/+*^ vs *Lrrk2*^*−/−*^, *p* < 0.001; *Lrrk2*^*+/+*^ vs *Lrrk2*^*GS/GS*^, *p* > 0.05; *Lrrk2*^*GS/GS*^ vs *Lrrk2*^*−/−*^, *p* < 0.001; Kruskal-Wallis test followed by Dunn’s multiple comparisons test). *Lrrk2*^*+/+*^ and *Lrrk2*^*−/−*^ astrocytes have similar overall volume occupied by lysosomal structures calculated as mean lysosomal number × mean lysosome volume (1.7 and 2.0 μm^3^/mm^2^). However, *Lrrk2*^*GS/GS*^ astrocytes showed half of the lysosomal global volume (1.0 μm^3^/mm^2^) compared to *Lrrk2*^*+/+*^ and *Lrrk2*^*−/−*^ astrocytes. To confirm our data, we analyzed the astrocytic endo-lysosomal compartment by immunofluorescence using Lamp2A as a marker of late endosomes/lysosomes. Also, with this approach, we revealed a significant reduction of the organelle number in *Lrrk2*^*−/−*^ and *Lrrk2*^*GS/GS*^ astrocytes (Fig. 4D–E; *Lrrk2*^*+/+*^ vs *Lrrk2*^*−/−*^, *p* < 0.001; *Lrrk2*^*+/+*^ vs *Lrrk2*^*GS/GS*^, *p* < 0.001; *Lrrk2*^*GS/GS*^ vs *Lrrk2*^*−/−*^, *p* < 0.01; one-way ANOVA Tukey’s multiple comparison test) associated with organelle enlargement in the *Lrrk2*^*−/−*^ cells (Fig. 4D–F; *Lrrk2*^*+/+*^ vs *Lrrk2*^*−/−*^, *p* < 0.001; *Lrrk2*^*+/+*^ vs *Lrrk2*^*GS/GS*^, *p* < 0.001; *Lrrk2*^*GS/GS*^ vs *Lrrk2*^*−/−*^, *p* < 0.001; Kruskal-Wallis test followed by Dunn’s multiple comparisons test).

To investigate whether lysosomal morphology impacts lysosomal pH, we compared primary astrocytes from the three genetic backgrounds using a ratiometric probe (RatioWork PDMPO), which labels acidic organelles and is independent from the endo-lysosomal content. The assay does not underline any differences between the three genotypes in the absence or in the presence of unlabeled α-syn PFFs (Fig. 4G). As a control, bafilomycin that dissipates endo-lysosomal pH causes a significant increase of the fluorescence ratio in all the conditions tested (Fig. 4G).

Next, the activation of the endo-lysosomal pathway was assessed in α-syn PFF-treated striatal astrocytes and untreated control cells by applying the neutral red assay. Neutral red is a non-ratiometric dye that is specifically retained by acidic vesicles [53] and it is frequently used as a reliable indicator of phagocytic activity [54, 55]. As expected, bafilomycin markedly decreases neutral red accumulation in all experimental conditions (Fig. 4H). In contrast, Neutral red staining is detected in cells in the absence of bafilomycin treatment by measuring absorbance at 540 nm in cell lysates. We did not report any statistical difference between the three genotypes in the absence of α-syn PFFs (under untreated condition) (Fig. 4H). However, PFF-stimulation induced increase of neutral red staining in astrocytes, indicating that phagocytosis efficiently occurs in *Lrrk2*^*+/+*^ and *Lrrk2*^*−/−*^ astrocytes but not in *Lrrk2*^*GS/GS*^ cells (Fig. 4H; *Lrrk2*^*+/+*^ vs *Lrrk2*^*+/+*^ PFFs, *p* < 0.001; *Lrrk2*^*−/−*^ vs *Lrrk2*^*−/−*^ PFFs, *p* < 0.001; *Lrrk2*^*GS/GS*^ vs *Lrrk2*^*GS/GS*^ PFFs, *p* < 0.05; one-way ANOVA Tukey’s multiple comparison test).

Collectively, these observations suggest that the G2019S mutation influences morphology and number of the endo-lysosomal vesicles but not the pH. However, the overall lysosomal degradation capacity appeared reduced in the presence of G2019S pathological mutation.

### LRRK2 Interacts with ANXA2

**Fig. 5:**
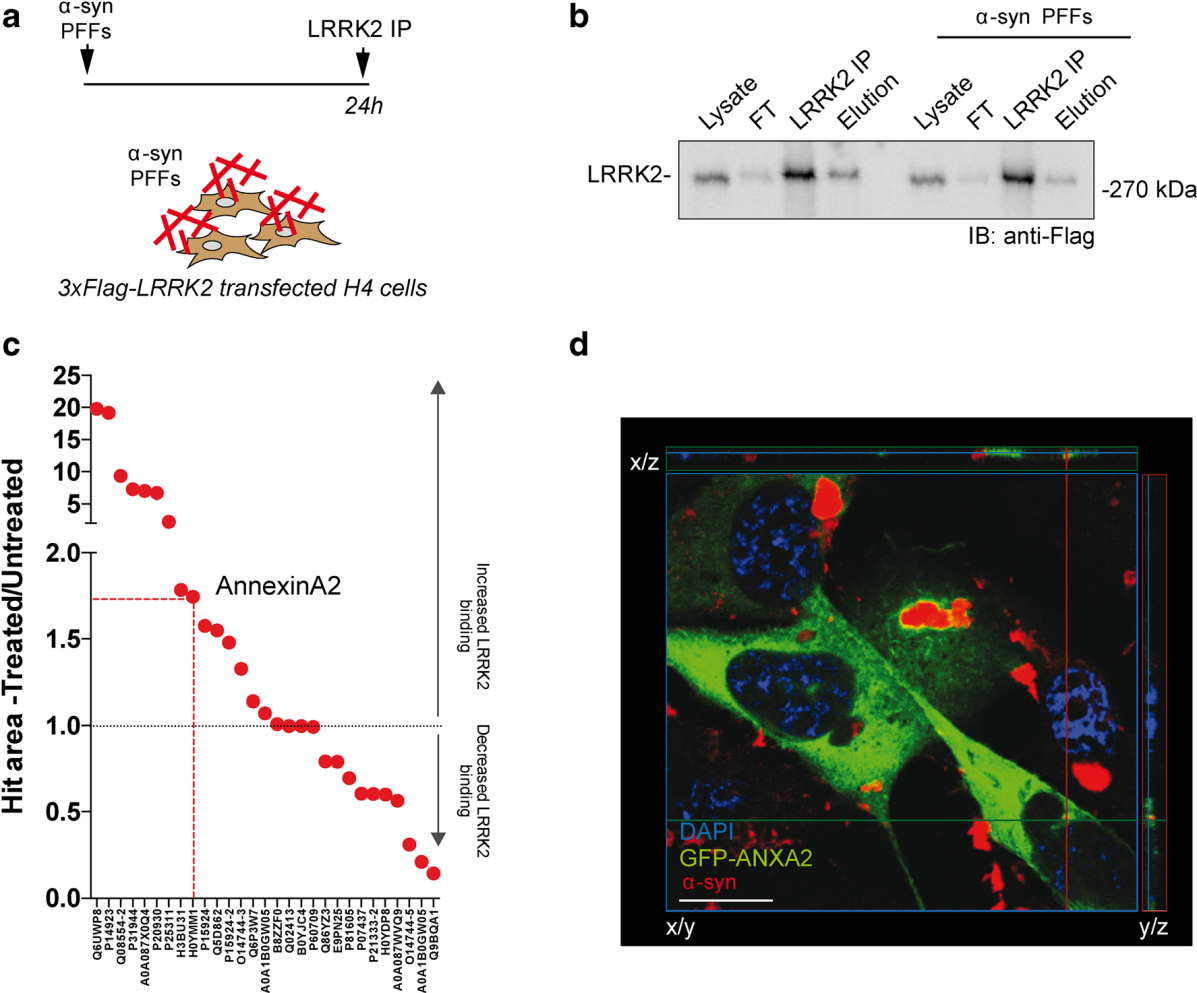
Characterization of LRRK2 interactoma in stimulated condition. **A** Schematic outline of the experimental setup. H4 cells were transfected using 3xFlag-LRRK2 encoding plasmid and, after 48 h post transfection, treated with 0.5 μM sonicated unlabeled α-syn PFFs for 24 h. LRRK2 was subsequently immunopurified using anti-Flag agarose beads (IP), eluted with Flag peptide (elution), and subjected to LC-MS/MS analysis. FT, flow-through; unbound LRRK2. **B** Western blot analysis showing LRRK2 expression, immunopurification, and elution in H4 cells in treated and basal conditions. **C** Relative quantification of LRRK2 interactome under treated and untreated conditions. The area of the precursor ions identified by LC-MS/MS analysis was used as a quantitative measure of the protein content. The ratio between the area of the precursor ions of untreated and treated samples (normalized by the content of LRRK2) was then considered to highlight proteins showing a different affinity for LRRK2 in the two conditions (*n* = 2). **D** H4 cells transfected with GFP-ANXA2 in α-syn PFF-treated condition verifying the proximity of internalized α-syn fibrils and transfected AnxA2. AnxA2-GFP (green), α-syn (red), DAPI (blue). Scale bar 30 μm

**Fig. 6:**
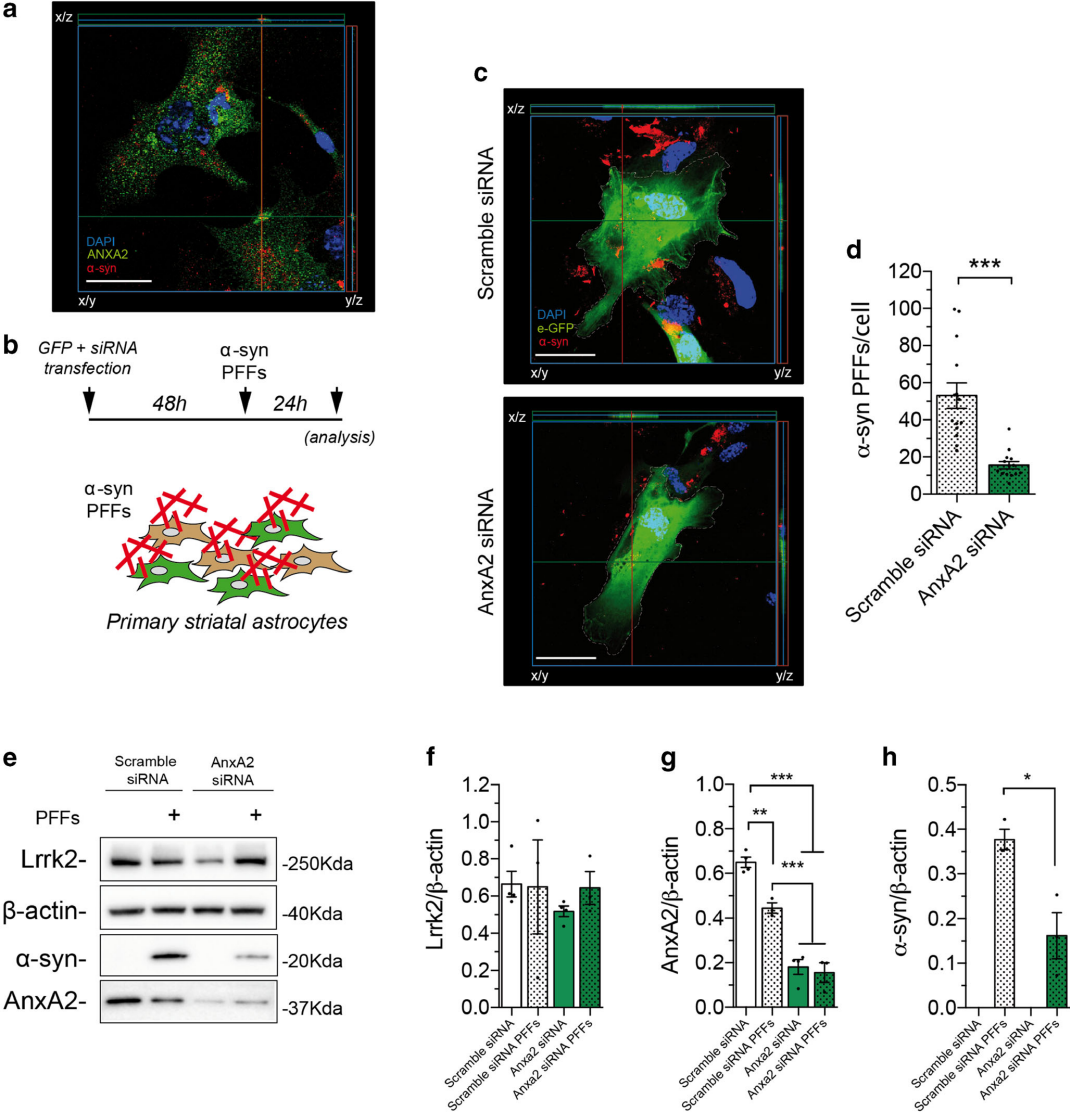
Investigation of AnxA2 function in astrocyte-mediated α-syn phagocytic clearance. **A** Unlabeled α-syn PFFs have been applied to primary astrocytes for 24 h. Projections verify the proximity of the internalized α-syn fibrils and endogenous AnxA2. Cell cytoskeleton (F-actin, cyano), α-syn (red), AnxA2 (green), DAPI (blue). Scale bar 20 μm. **B** Schematic outline of the experimental setup. Cells were transfected using 3xFlag-GFP-encoding plasmid together with scramble or AnxA2 siRNA. 48 h post transfection cells were treated with 0.5 μM sonicated α-syn PFFs for 24 h and imaged using confocal microscopy. **C** Representative images of primary striatal astrocytes transfected with scramble or Anxa2 siRNA together with a GFP-encoding plasmid in α-syn PFF-treated condition. Projections verify the proximity of the internalized α-syn fibrils; GFP (green), α-syn (red), DAPI (blue). Scale bar 30 μm. **D** Four images per cell culture were analyzed (*n* = 3). Quantifications of α-syn PFFs fluorescent-positive puncta were performed using ImageJ (ComDet plug-in). **E** Western blot analysis of primary striatal astrocyte lysates transfected with scramble and AnxA2 siRNA under basal and PFF-treated conditions. Anti-Lrrk2, anti-α-syn, and anti-AnxA2 antibodies have been employed. **F**, **G**, **H** Quantification of band intensity was performed using ImageJ and normalized by β-actin (*n* = 3). Statistical analysis in **D** was made by Kruskal-Wallis test followed by Dunn’s multiple comparisons test. Statistical analysis in **F**, **G**, and **H** was performed with an unpaired *t* test or one-way ANOVA followed by Tukey’s multiple comparisons test. *p ≤ 0.05, **p ≤ 0.01, ***p ≤ 0.001

To identify LRRK2-specific effectors of astrocyte-mediated α-syn PFF clearance using an unbiased approach, we performed a high-throughput screening of candidate protein-protein interaction (PPI) partners via affinity purification (AP) coupled with tandem mass spectrometry. Specifically, we processed LRRK2-immunoprecipitated binders by liquid chromatography–mass spectrometry (LC-MS) from H4 cells (Fig. 5A). LRRK2 was immunopurified using anti-Flag agarose beads and eluted from the resin to exclude contaminants with high affinity for the resin. With this analysis, we compared LRRK2 interactome in PFF-treated and untreated samples. In Fig. 5B, we show the LRRK2 immunopurification steps in the two conditions. For each interactor, we calculated the ratio between the chromatographic area relative to the peptides belonging to the proteins detected in treated versus non-treated conditions. The value was normalized by the peak area of immunoprecipitated LRRK2 in the two experimental conditions and reported in Fig. 5C. To select possible players in LRRK2-mediated α-syn PFF clearance, we focused our attention on (i) hits that are recruited by LRRK2 upon treatment, (ii) functional relevance, and (iii) expression in glial cells (Supplementary Figure 6A, B). Out of the mass spectrometry hits recovered from two independent replicates, annexin A2 (human ANXA2) displayed increased affinity for LRRK2 upon α-syn PFF treatment. Intriguingly, ANXA2 is a phospholipid-binding protein that intervenes in phagocytic processes at multiple levels [56–62]. Specifically, it was reported that *AnxA2* deficits are linked to a decreased endocytosis and particle internalization [56, 63, 64]. Moreover, ANXA2 is almost exclusively expressed in glial cells in the brain [65]. We first validated the role of ANXA2 in regulating exogenous fibrillar α-syn internalization. In H4 astrocytic cells, GFP-transfected ANXA2 shows a diffuse cytoplasmic distribution, while it re-localizes around α-syn upon treatment with exogenous fibrils (Fig. 5D and Supplementary Figure 8). As reported for ectopically expressed GFP-ANXA2 in H4 cells (Fig. 5D), endogenous mouse astrocytic AnxA2 localizes into subcellular puncta in the near proximity of internalized PFF particles (Fig. 6A). To evaluate the involvement of mouse AnxA2 in astrocyte-mediated α-syn PFF phagocytic clearance at the endogenous level, we acutely downregulated *AnxA2* in primary striatal astrocytes using siRNA. Around 60% of *AnxA2* downregulation was achieved in *AnxA2* siRNA-transfected astrocytes compared to controls (Supplementary Figure 9A–C). *AnxA2* downregulation did not significantly affect LRRK2 protein levels in transfected astrocytes (Supplementary Figure 9A–B). To image siRNA recipient cells, we co-transfected primary striatal astrocytes with *AnxA2* siRNA and a GFP-encoding plasmid. As represented in Fig. 6B, we acquired images after 24 h of PFF treatment. Quantification of internalized α-syn-positive puncta in GFP-positive cells revealed that *AnxA2* downregulation decreases the amount of intracellular deposits per cell compared to scramble transfected controls (Fig. 6C–D; *p* < 0.001; Kruskal-Wallis test followed by Dunn’s multiple comparisons test). As an additional control for AnxA2 downregulation at single-cell resolution, we stained transfected GFP-positive astrocytes for the endogenous protein. Quantification of AnxA2 puncta upon PFF treatment is significantly reduced in *AnxA2* siRNA versus control (Supplementary Figure 9D–E; *p* < 0.001; unpaired *t* test). To validate our findings, we also evaluated intracellular α-syn PFFs by western blot analysis upon *AnxA2* downregulation (Fig. 6E). As already shown in Supplementary Figure 9, siRNA transfection in primary striatal astrocytes does not affect LRRK2 protein level, neither in treated nor untreated conditions (Fig. 6E–F). Again, *AnxA2* downregulation was successfully achieved and maintained after PFF treatment (Fig. 6E–G; scramble siRNA vs *AnxA2* siRNA +/− PFFs, *p* < 0.001; scramble siRNA + PFFs vs *AnxA2* siRNA +/− PFFs, *p* < 0.001; one-way ANOVA followed by Tukey’s multiple comparisons test). A slight but significantly decreased level of AnxA2 was also revealed upon PFF exposure in the scramble siRNA astrocytes (Fig. 6E–G; scramble siRNA vs scramble siRNA + PFFs, *p* < 0.01; one-way ANOVA followed by Tukey’s multiple comparisons test). However, our results showed that the amount of accumulated α-syn significantly decreased by comparing lysates obtained from *AnxA2* siRNA-transfected astrocytes versus control (Fig. 6E–H; scramble siRNA + PFFs vs *AnxA2* siRNA + PFFs, *p* < 0.01; unpaired *t* test).

Overall, these findings suggest a functional link between Lrrk2 and AnxA2 in astrocyte-mediated exogenous α-syn clearance and that *AnxA2* deficits influence the astrocytic ability to store α-syn aggregates.

### G2019S Lrrk2 Striatal Astrocytes Display AnxA2 Deficits

**Fig. 7:**
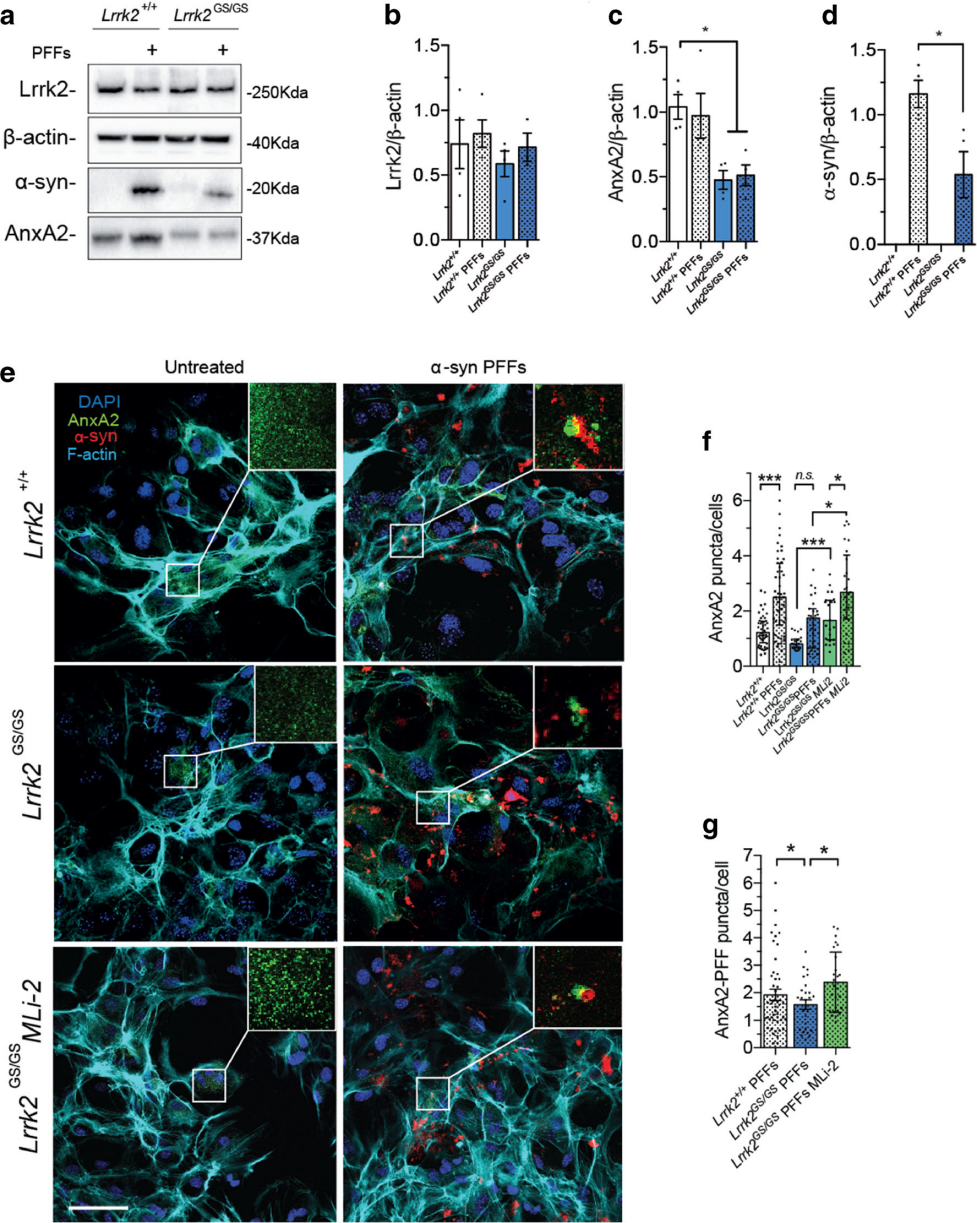
Analysis of AnxA2 function in G2019S primary striatal astrocytes at endogenous level. **A** Western blot analysis of primary striatal astrocyte lysates under PFF-treated and basal conditions using anti-Lrrk2, anti-α-syn, and anti-AnxA2 antibodies. **B**, **C**, **D** Quantification of band intensity was performed using ImageJ and normalized by β-actin (*n* = 4). **E** Representative images of *Lrrk2*^*+/+*^ and *Lrrk2*^*GS/GS*^ astrocytes treated or not with α-syn PFFs and stained with anti-AnxA2 (green), anti-α-syn (red), F-actin (cyano), and cell nuclei with DAPI (blue). MLi-2-treated *Lrrk2*^*GS/GS*^ astrocytes are shown in the bottom panels. Scale bar 20 μm. Insets show a close-up of α-syn inclusions and re-localized AnxA2. **F**, **G** Eight images per cell culture were analyzed (*n* = 3). Quantifications of Anxa2 puncta and AnxA2-α-syn PFFs proximity were performed using ImageJ. Statistical analysis in **F** and **G** was made by Kruskal-Wallis test followed by Dunn’s multiple comparisons test. Statistical analysis in **B**, **C**, and **D** was performed with unpaired *t* test or one-way ANOVA followed by Tukey’s multiple comparisons test. *p ≤ 0.05, **p ≤ 0.01, ***p ≤ 0.001

To determine whether Lrrk2 impacts α-syn PFF clearance through AnxA2, we measured AnxA2 protein content in treated versus untreated striatal primary astrocytes. Here, we focused our analysis on *Lrrk2*^*+/+*^ versus *Lrrk2*^*GS/GS*^ genotype*.* Cell lysates from cultured striatal astrocytes were subjected to western blot and AnxA2 endogenous expression was assayed. As shown in Fig. 7A, AnxA2 level is significantly decreased in *Lrrk2*^*GS/GS*^ astrocytes both under basal and treated conditions compared to wild-type cells (Fig. 7A and C; *Lrrk2*^*+/+*^ vs *Lrrk2*^*GS/GS*^ +/− PFFs, *p* < 0.05; one-way ANOVA followed by Tukey’s multiple comparisons test). In agreement with our data reported in Fig. 6, AnxA2 downregulation in G2019S *Lrrk2*^*GS/GS*^ astrocytes is associated with a significant decrease in intracellular α-syn deposits (Fig. 7A and D; *Lrrk2*^*+/+*^ *+* PFFs vs *Lrrk2*^*GS/GS*^ + PFFs, *p* < 0.05; unpaired *t* test). However, the LRRK2 protein level was not affected in *Lrrk2*^*GS/GS*^ astrocytes in neither treated nor untreated conditions (Fig. 7A–B). We then evaluated AnxA2 localization under basal condition and upon α-syn PFFs treatment in *Lrrk2*^*GS/GS*^ astrocytes, compared to control astrocytes (Fig. 7E). As already reported by others [57] and shown here for H4 astrocytic cells, ANXA2 is homogenously distributed within the cytoplasm under basal conditions, but accumulates into discrete puncta upon stimuli. Using ImageJ, we quantified ANXA2 fluorescent-positive puncta in control and α-syn PFF–exposed astrocytes of the two genotypes. Our results show that ANXA2 re-localization takes place in both *Lrrk2*^*+/+*^ and *Lrrk2*^*GS/GS*^ astrocytes, while it appeared significantly decreased in cells harboring the pathogenic mutation (Fig. 7F; *Lrrk2*^*+/+*^ vs *Lrrk2*^*+/+*^ PFFs, *p* < 0.001; *Lrrk2*^*GS/GS*^ vs *Lrrk2*^*GS/GS*^ PFFs, *p* > 0.05; Kruskal-Wallis test followed by Dunn’s multiple comparisons test). To assess whether the pathological effect of Lrrk2 G2019S is mediated by the enhanced kinase activity, we treated *Lrrk2*^*GS/GS*^ primary astrocytes with MLi-2, a highly specific and selective LRRK2 inhibitor [66]. Upon MLi-2 treatment, AnxA2 is significantly increased at basal level in *Lrrk2*^*GS/GS*^ primary astrocytes compared to untreated cells (Fig. 7F; *Lrrk2*^*GS/GS*^ vs *Lrrk2*^*GS/GS*^ MLi-2, *p* < 0.001; Kruskal-Wallis test followed by Dunn’s multiple comparisons test). Moreover, AnxA2 re-localization into puncta is significantly enhanced in MLi-2 treated *Lrrk2*^*GS/GS*^ primary astrocytes upon PFFs addition (Fig. 7F; *Lrrk2*^*GS/GS*^ MLi-2 vs *Lrrk2*^*GS/GS*^ PFFs MLi-2 *p* < 0.01; Kruskal-Wallis test followed by Dunn’s multiple comparisons test). Coherently, statistical significance is also detected between *Lrrk2*^*GS/GS*^ primary astrocytes treated with PFFs in the presence and in the absence of MLi-2 (Fig. 7F; *Lrrk2*^*GS/GS*^ PFFs vs *Lrrk2*^*GS/GS*^ PFFs MLi-2, *p* < 0.01; Kruskal-Wallis test followed by Dunn’s multiple comparisons test). We then quantified α-syn deposits associated with ANXA2 puncta within each cell by measuring particle proximity using ImageJ. We observed that less α-syn intracellular inclusions are found in close proximity to ANXA2 in *Lrrk2*^*GS/GS*^ striatal astrocytes compared to *Lrrk2*^+/+^ controls (Fig. 7E and G; *Lrrk2*^*+/+*^ vs *Lrrk2*^*GS/GS*^, *p* < 0.01; Kruskal-Wallis test followed by Dunn’s multiple comparisons test). The addition of MLi-2 inhibitor in astrocytes harboring the pathological mutation reverts the observed phenotype (Fig. 7E; *Lrrk2*^*GS/GS*^ PFFs vs *Lrrk2*^*GS/GS*^ MLi-2, *p* < 0.01; Kruskal-Wallis test followed by Dunn’s multiple comparisons test).

Overall, our results show that the ANXA2 level is diminished in *Lrrk2*^*GS/GS*^ astrocytes and this phenomenon is associated with a decreased number of intracellular α-syn deposits. Importantly, pharmacological inhibition of Lrrk2 kinase activity fully reverts the mutant phenotype.

## Discussion

Aggregated α-syn is the main constituent of the intra-neuronal and intra-glial proteinaceous inclusions found in PD [4, 7]. Compelling evidence suggests that cell-to-cell transmission of the α-syn aggregates results in the anatomical spread of the disease [67–73]. The intercellular transfer can occur through different mechanisms, including tunneling nanotubes, exosomes, and secretion of free pathological α-syn aggregates [9, 71, 74–76]. Following secretion, α-syn aggregates are sequestered from the extracellular space by neighboring cells [71–73, 76–78]. Astrocytes, which are the major glial cell type, are known to ingest particularly large amounts of aggregated α-syn that are then intracellularly stored, rather than degraded [9, 32]. We have previously shown that the accumulation of aggregated α-syn in human astrocytes disrupts their lysosomal machinery, induces cell-to-cell transfer between astrocytes, and promotes T cell activation [8, 9]. However, the exact role of astrocytes in PD and the particular molecular mechanisms that impact their clearing capacity remain elusive. In the last few years, several reports have suggested that pathogenic and functional mutations in LRRK2 influence the biology of human and murine astrocytes at multiple levels both in vivo and in vitro [24, 79–83]. In addition, LRRK2 is emerging as a key player in the clearance of extracellular particles in macrophages and monocytes, with its involvement both in internalization step and downstream in vesicle maturation and sorting [22–24, 26, 27]. However, no investigation has focused on the role of LRRK2 in astrocyte-mediated α-syn clearance.

We started our investigation by studying the effect of *Lrrk2* in α-syn clearance over time using an astrocytic culture model [29, 37–39]. Our study revealed that the most common pathogenic mutation in LRRK2, G2019S, negatively regulates the amount of engulfed fibrillar α-syn in astrocytes. In agreement with our evidence, it has recently been proposed that LRRK2 kinase activity blocks micropinocytosis in phagocytes through the phosphorylation of Rab10 [27]. Analyzing the differently sized α-syn inclusions separately revealed that the reduced α-syn accumulation in *Lrrk2*^*GS/GS*^ astrocytes was predominantly driven by the small α-syn inclusions. However, this effect normalized over time (in the absence of further α-syn PFFs in the culture medium) and could be explained by the attempt of astrocytes to degrade the engulfed material, but being inefficient they rather store the ingested α-syn, which we have also seen in previous studies [9, 32]. Indeed, the amount of accumulated α-syn 6 days after the exposure to α-syn PFFs increased in all three genotypes, with a more pronounced effect observed in the presence of the pathological mutation. This increase cannot be attributed to more α-syn being taken up by the astrocytes, since the cells were rinsed extensively after the 24-h exposure to α-syn PFFs. Instead, the reason for the increase was the redistribution of α-syn aggregates inside the cell. Over the 6 days, the intracellular α-syn deposits are brought closer together in the region around the nucleus, which results in larger inclusions with stronger fluorescence signal. The larger area measurements in combination with an unchanged particle count indicates the formation of bigger aggregates overall. Also, α-syn particles, previously below the detection limit, may have clustered and thus become detectable at a later time point.

In the neural stem cell–based astrocyte model, apoptotic cells are naturally produced during the differentiation phase, which are engulfed and stored by the astrocytes. The reason why pyknotic cell nuclei and large α-syn deposits appear in close proximity inside the astrocytes is that the cells direct the ingested material to the same cellular compartments, or “storage dumps.” This is a phenomenon that we have previously demonstrated, in different astrocytic culture systems, following α-syn or amyloid-beta exposure [9, 31]. Although the storage of cell corpses and protein aggregates is stressful for the astrocytes, the number of viable astrocytes during the experimental timeframe has been shown to remain constant [31]. We have previously observed that engulfed α-syn oligomers and the lysosomal marker Lamp-1 only temporarily co-localize [9, 32]. Thus, the persisting α-syn deposits could be a sign of an overwhelmed degradation system, which has been shown to result in cytoplasmic aggregates known as aggresomes as well as inclusion bodies [84]. This suggests that astrocytes try to degrade the engulfed α-syn, but in the end fail to do so and rather store and to some extent transfer the engulfed material to neighboring astrocytes.

Astrocytes that populate the striatum are relevant to PD pathology since they are in close proximity to dopaminergic terminals of the SNpc and it is unknown how they respond to neuronal-released α-syn. Here, we confirmed that the G2019S pathogenic mutation in *Lrrk2* reduces the amount of internalized α-syn in primary mouse striatal astrocytes without impairing the pH and the flow of particles through the endo-lysosomal system. However, Lrrk2 pronouncedly changes the architecture of the late-endosome/lysosomal organelles in striatal astrocytes. By TEM, we showed that the genetic ablation of *Lrrk2* causes an enlargement of lysosomal structures in primary striatal astrocytes. This finding is in agreement with several other published observations of kidney and lung tissues [20, 85]. The change in lysosomal size was however compensated by an overall decrease in lysosomal structures, as shown for both *Lrrk2*^*−/−*^ primary striatal astrocytes and for astrocytes in *Lrrk2*^*−/−*^ striatum brain sections. A similar trend has been observed by measuring Lamp2A endo-lysosomal–positive structures. The pathogenic G2019S mutation in *Lrrk2* induces an endo-lysosomal shrinkage associated with a decreased number of organelles in primary striatal astrocytes, both when quantifying lysosomal-like structures with TEM and Lamp2A-positive structures using confocal imaging. Similar morphological data has been observed in G2019S PD patient–specific human neuroepithelial stem cells and in primary cortical neurons from *Lrrk2*^*GS/GS*^ mice, using close or identical experimental settings [19, 86]. In contrast to the above findings, Henry et al. indicated enlarged lysosomes in cultured cortical astrocytes overexpressing G2019S human LRRK2 compared to wild-type astrocytes, without discriminating between the contribution of the pathogenic mutation and the LRRK2 level [12]. The overall volume occupied by lysosomal-like structures indicates that striatal *Lrrk2*^*GS/GS*^ astrocytes present an intrinsic, basal deficit in the endo-lysosomal holding capacity (half total volume) with respect to the wild-type counterpart although maintaining identical pH. Contrary to our imaging results, we did not detect any differences between genotypes by labeling acidic vesicles with neutral red solution at the basal level, suggesting that a bulk approach might not be sufficiently sensitive. However, astrocytes displayed increased staining of the acidic compartment using neutral red solution after 24 h of PFF treatment, in agreement with enhanced phagocytic activity. *Lrrk2*^*GS/GS*^ cells did not show any intensification of neural red staining suggesting a possible impairment in their ability to ingest extracellular α-syn. In phagocytes, it was recently reported that the endo-lysosomal compartment undergoes adaptation, remodeling, and expansion during particle internalization through an enhanced translation [87]. In this regard, our findings might suggest that striatal astrocytes harboring the G2019S mutation in *Lrrk2* also fail to adapt and expand their endo-lysosomal system when they are stimulated to phagocytize, resulting in α-syn-defective internalization. This concept could explain why *Lrrk2*^*−/−*^ astrocytes, which possess an overall volume occupied by the degradative organelles almost comparable to the wild-type, do not show any defects in terms of PFF internalization.

For the first time, our study identifies ANXA2 as a novel player in α-syn clearance in astrocytic cells. ANXA2 is an actin-binding protein that modulates many intracellular trafficking events, via the regulation of actin polymerization dynamics. Specifically, ANXA2 is recruited to the plasma membrane, during the formation of the phagocytic cup [56], and assists endosomes upon particle internalization by preventing destabilization [57]. Of note, several papers reported the involvement of ANXA2 in autophagy [58–61], a degradative process highly interconnected with the clearance of pathogens and aggregated proteins, including α-syn [62]. Indeed, multiple lines of evidence indicate that *AnxA2* knockdown or knock-out prevents endocytic transport beyond early endosomes, interferes with particle phagocytic transport, and causes endosomal ultrastructural impairments [56, 88, 89]. Relevant for diseases associated with the accumulation of aggregated proteins, ANXA2 expression is enhanced at the cell periphery in reactive astrocytes positioned in close proximity to senile plaques and degenerating neurons of Alzheimer’s disease human post mortem brains [90]. This immunohistological data suggest that ANXA2 might be important for the clearance of extracellular toxic proteinaceous material. Indeed, we demonstrated that endogenous *AnxA2* downregulation negatively impacts exogenous α-syn clearance in primary striatal astrocytes as shown by the decreased amount of intracellular α-syn deposits as well as unfolded α-syn in the lysates. Of note, ANXA2 enhanced its affinity for LRRK2 upon α-syn PFFs treatment in astrocytic-like cells pointing to a functional interaction between the two proteins. Astrocytes expressing pathogenic Lrrk2 displayed a significant AnxA2 deficit at the protein level followed by a reduction in intracellular α-syn-positive puncta and α-syn in the lysates. Endogenous AnxA2 in *Lrrk2*^*GS/GS*^ astrocytes has impaired ability to regulate (i) intracellular re-localization into puncta and (ii) proximity to internalized α-syn particles. AnxA2 downregulation can also explain the endo-lysosomal shrinkage showed in *Lrrk2*^*GS/GS*^ astrocytes. Of note, AnxA2 deficits in *Lrrk2*^*GS/GS*^ astrocytes are completely reverted by a long-term application of Lrrk2 kinase inhibitors. How LRRK2 kinase activity regulates the ANXA2 level in astrocytes and the impact of the G2019S mutation in this process needs to be explored in the future. Aggregated α-syn is engulfed by cells by different molecular processes, including receptor-mediated endocytosis and phagocytosis that probably differ according to particle dimension as well as cell type [91–93]. Our study reveals that the pathogenic G2019S mutation primarily affects the clearance of exogenous small α-syn inclusions, possibly by reducing internalization rather than enhancing degradation. In this regard, future studies will be needed to address the molecular mechanism of PFF handling mediated by ANXA2.

## Conclusions

Being the most numerous glial cell type in the central nervous system, astrocytes play a major role in orchestrating brain homeostasis. Recently, it has been shown that they uptake exogenous fibrillar α-syn. Yet, their exact role in PD pathology remains elusive. In the present study, we identify astrocytes as possible players in LRRK2-mediated PD pathology. Taken together, our results demonstrate that LRRK2 impacts astrocyte-mediated α-syn clearance. Astrocytes harboring the most common pathogenic PD-linked mutation G2019S in *Lrrk2* display a reduction of internalized α-syn as well as an impairment of their endo-lysosomal capacity. This impairment is associated with deficits in the amount of AnxA2, a novel player shown to be involved in the clearance process that is highly expressed by glial cells. Our data offer a better understanding of the molecular mechanism behind impaired α-syn clearance in G2019S astrocytes and highlight astrocytes as a promising target in the context of LRRK2-mediated PD. Indeed, aberrant mechanisms of α-syn clearance might contribute to α-syn stagnation in the extracellular space thus enhancing toxicity. Hence, to restore/enhance astrocyte-mediated α-syn phagocytic clearance could be a valuable therapeutic approach.

## Supplementary Information

ESM 1 (DOCX 27037 kb)

## Abbreviations

AnxA2: Annexin A2
AP: Affinity purification
α-syn: Alpha-synuclein
bFGF: Basic fibroblast growth factor
BME: Basal Medium Eagle
BSA: Bovine serum albumin
DMEM: Dulbecco’s Modified Eagle Medium
DPBS: Dulbecco’s Phosphate Buffered Saline
DTT: Dithiothreitol
EGF: Epidermal growth factor
FBS: Fetal bovine serum
FDR: False discovery rate
GFAP: Glial fibrillary acidic protein
GFP: Green fluorescent protein
HRP: Horseradish peroxidase
LAMP: Lysosome-associated protein
LC-MS: Liquid chromatography–mass spectrometry
LRRK2: Leucine-rich repeat kinase 2
NGS: Normal goat serum
PD: Parkinson’s disease
PEI: Polyethylenimine
PFA: Paraformaldehyde
PFFs: Pre-formed fibrils
PPI: Protein-protein interaction
ROI: Region of interest
RT: Room temperature
TEM: Transmission electron microscopy
