# Supplementary material for: Parkinson’s Disease–Associated LRRK2 Interferes with Astrocyte-Mediated Alpha-Synuclein Clearance
Source: Mol Neurobiol. 2021 Feb 24;58(7):3119–40. doi: 10.1007/s12035-021-02327-8 (PMC8257537; doi:10.1007/s12035-021-02327-8)
Supplement: Supplementary file 1 — (DOCX 27037 kb) [file 12035_2021_2327_MOESM1_ESM.docx]

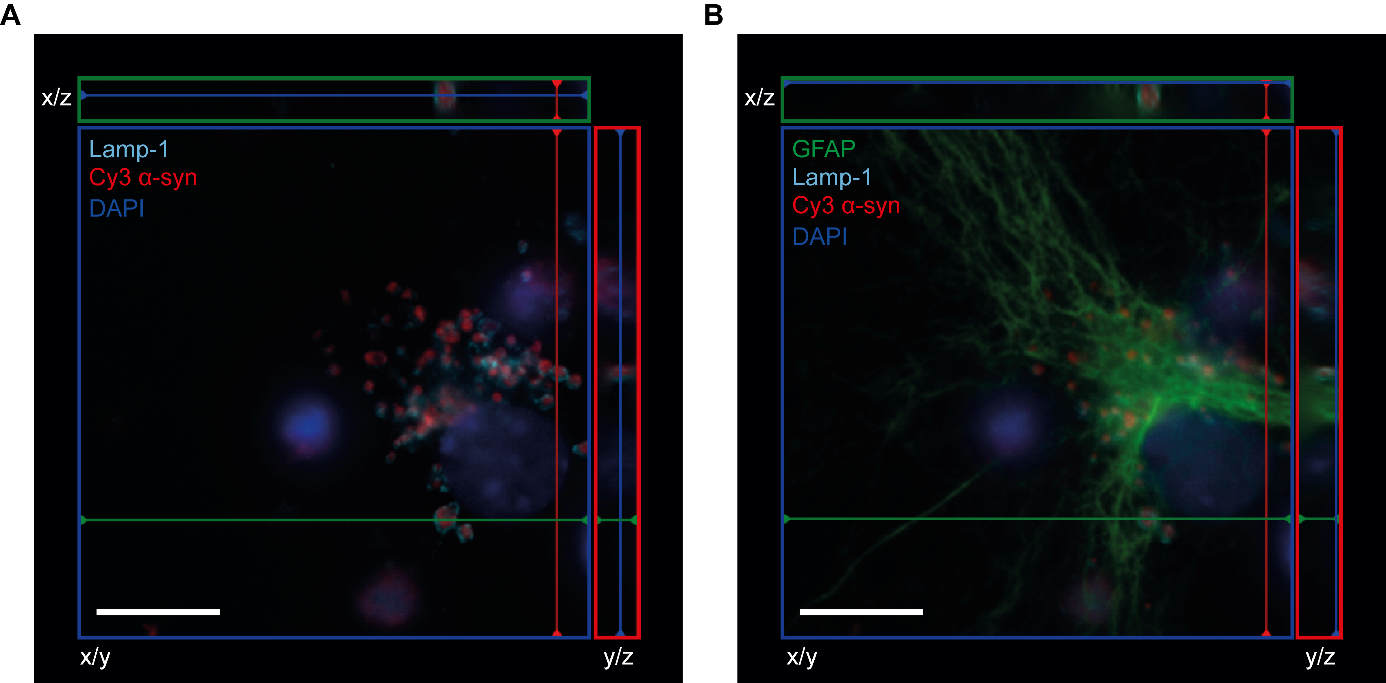


**Supplementary Figure 1** Ingested Cy3 α-syn was found in vesicles expressing the endo-lysosomal marker Lamp-1. **A and B)** Representative image of a *Lrrk2*^+/+^ astrocyte derived from embryonic, cortical stem cells showing Lamp‑1 staining surrounding Cy3 α-syn deposits. Lamp‑1 (light blue), Cy3 α-syn (red) and nuclei (DAPI, blue), astrocyte (GFAP, green). Scale bars = 10 µm (A and B).


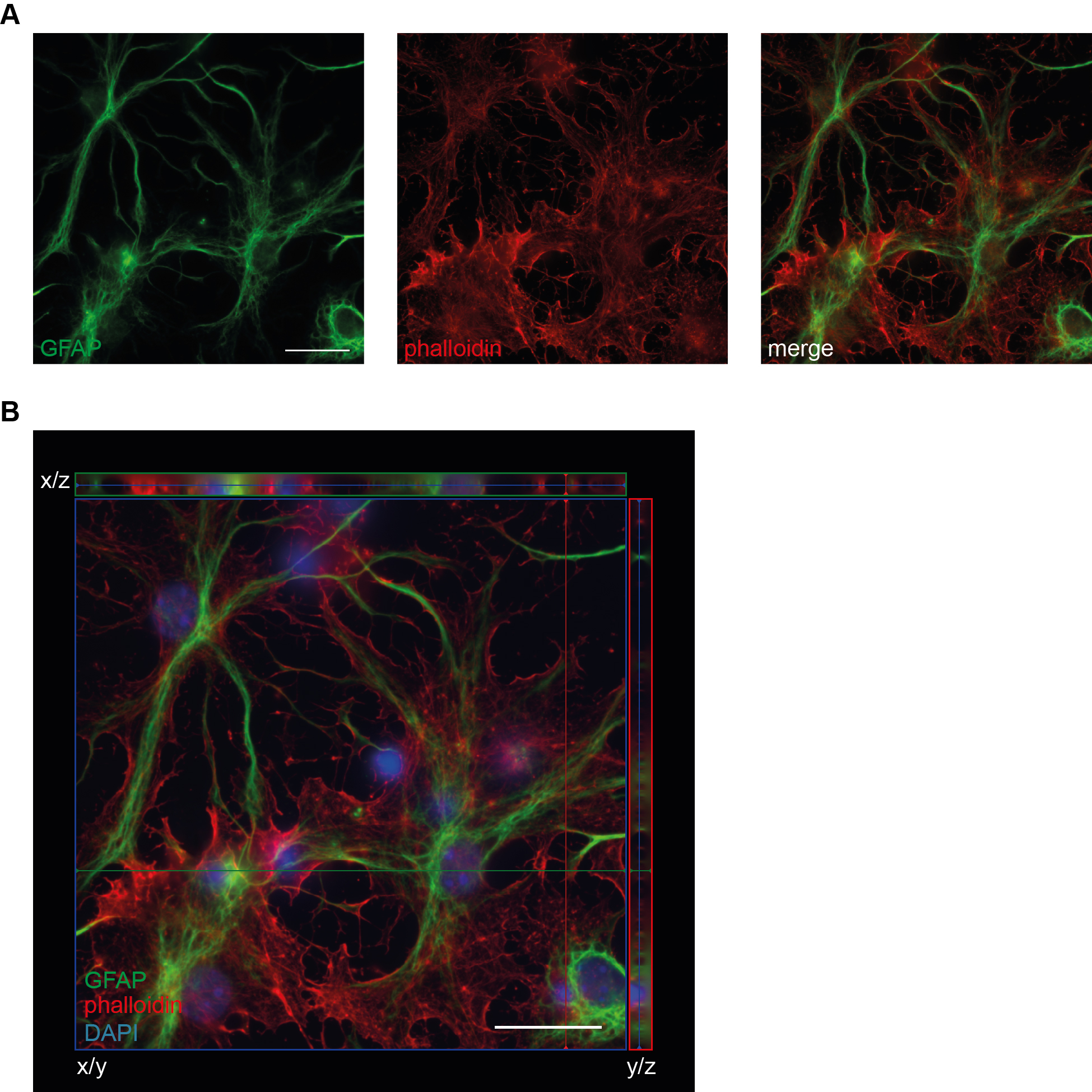


**Supplementary Figure 2** Characterization of GFAP and phalloidin stainings in astrocytes derived from embryonic, cortical stem cells. **A)** Representative images stained for astrocytes (GFAP, green) and actin (phalloidin, red). These images demonstrate that GFAP, as part of the cytoskeleton, only comprises a portion of the cell, the perimeter of which is shown by the actin stain. **B)** GFAP and actin staining enclosed areas with condensed DAPI staining and thus verified the internal localization of pyknotic cell nuclei in astrocytes. Astrocytes (GFAP, green), actin (phalloidin, red), nuclei (DAPI, blue). Scale bars = 20 µm (A and B).


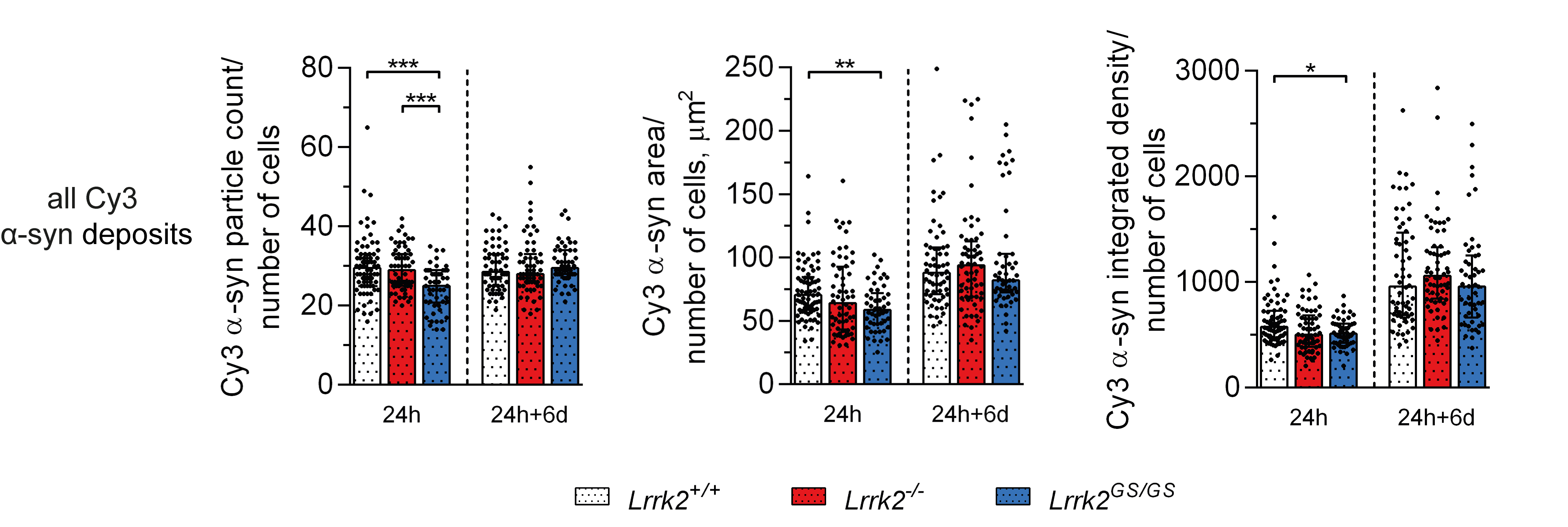


**Supplementary Figure 3** Analysis of all Cy3 α-syn inclusions combined. Quantifications of Cy3 α-syn particle count, total area and integrated density were performed using ImageJ. For both time-points, ten images per independent cell culture (24 h: *Lrrk1*^+/+^ n = 7, *Lrrk2*^-/-^ n = 6 and *Lrrk2*^GS/GS^ n = 5; 24 h + 6 d: *Lrrk1*^+/+^ n = 6, *Lrrk2*^-/-^ n = 6 and *Lrrk2*^GS/GS^ n = 5) were analyzed and reported. For each time-point, the statistical analysis was performed with the Kruskal-Wallis test followed by Dunn’s multiple comparisons test, since the data did not follow Gaussian distribution for all groups; * p ≤ 0.05 ** p ≤ 0.01 *** p ≤ 0.001. Area and integrated density measurements showed a 1.5 to 2-fold increase at the later time-point, while the number of particles counted did not change (apart for *Lrrk2^GS/GS^*, which showed a 1.2-fold change increase).


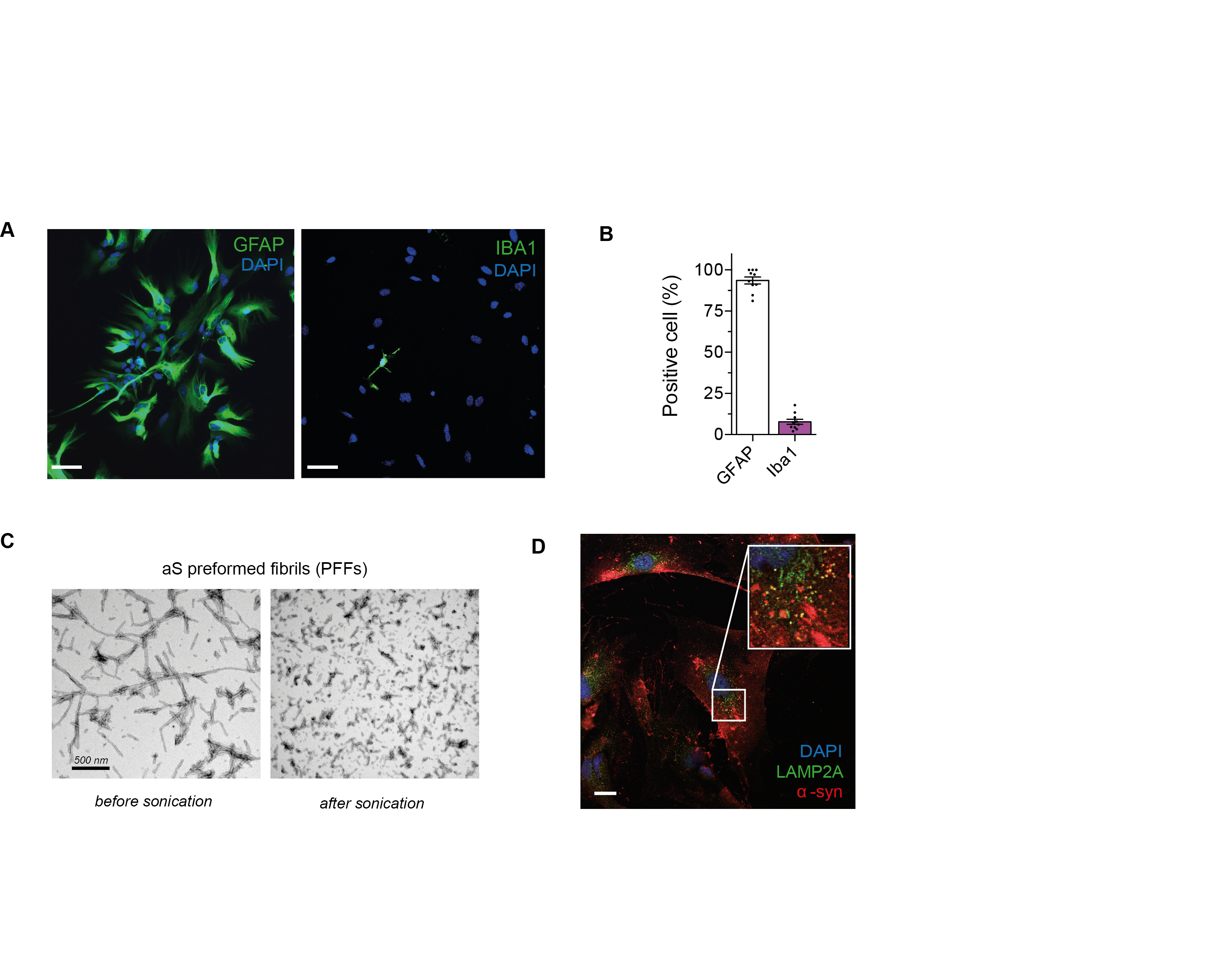


**Supplementary Figure 4** Characterization of striatal astrocytes. **A)** Representative fluorescence microscopy images, stained for astrocytes (GFAP, green) and microglia (Iba1, green). Scale bar 50 µm. **B)** Quantification of GFAP and Iba1 positive cells. The quantification demonstrates that isolation protocol for primary striatal astrocytes leads to a population 94% ± 2 astrocytes and 7% ± 2 microglia. Scale bar 50 µm **C)** Representative TEM images of pre- and post-sonicated α‑syn PFFs. Scale bar 500 nm. **D)** Fluorescence microscopy image of mouse primary striatal astrocytes treated with α‑syn PFFs (red) and stained for Lamp2A (green). Scale bar 5 µm.

**Supplementary Figure 5** SNARF-1 calibration. Free SNARF and sonicated SNARF-labeled α‑syn PFFs were resuspended using buffers at different pH and the emission spectra were acquired within 520-660 nm range upon laser excitation at 488 nm using a fluorimeter device. The value ratio 550/630 nm is reported in the graph.

**
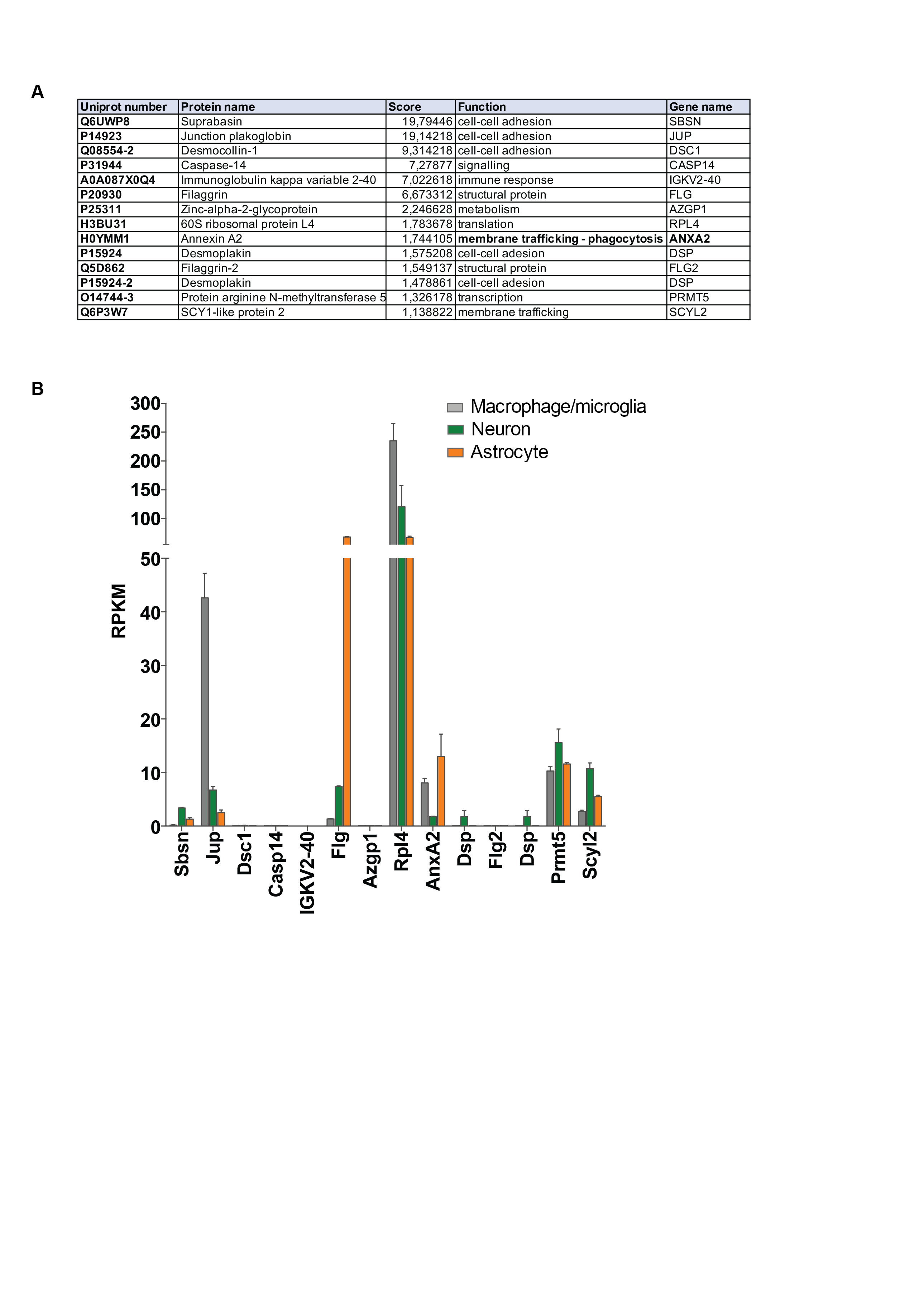
**

**Supplementary Figure 6** Selection criteria for possible players in LRRK2-mediated α-syn PFF clearance. Hits that increase LRRK2 binding upon treatment are listed in **A** with the reported cellular function. In **B**, transcriptomic data showing the expression of mouse genes in astrocytes, neurons and microglia/macrophages (<http://www.brainrnaseq.org/>) (1). Abbreviation: RPKM, reads per kilobase of transcript per million mapped reads.

**
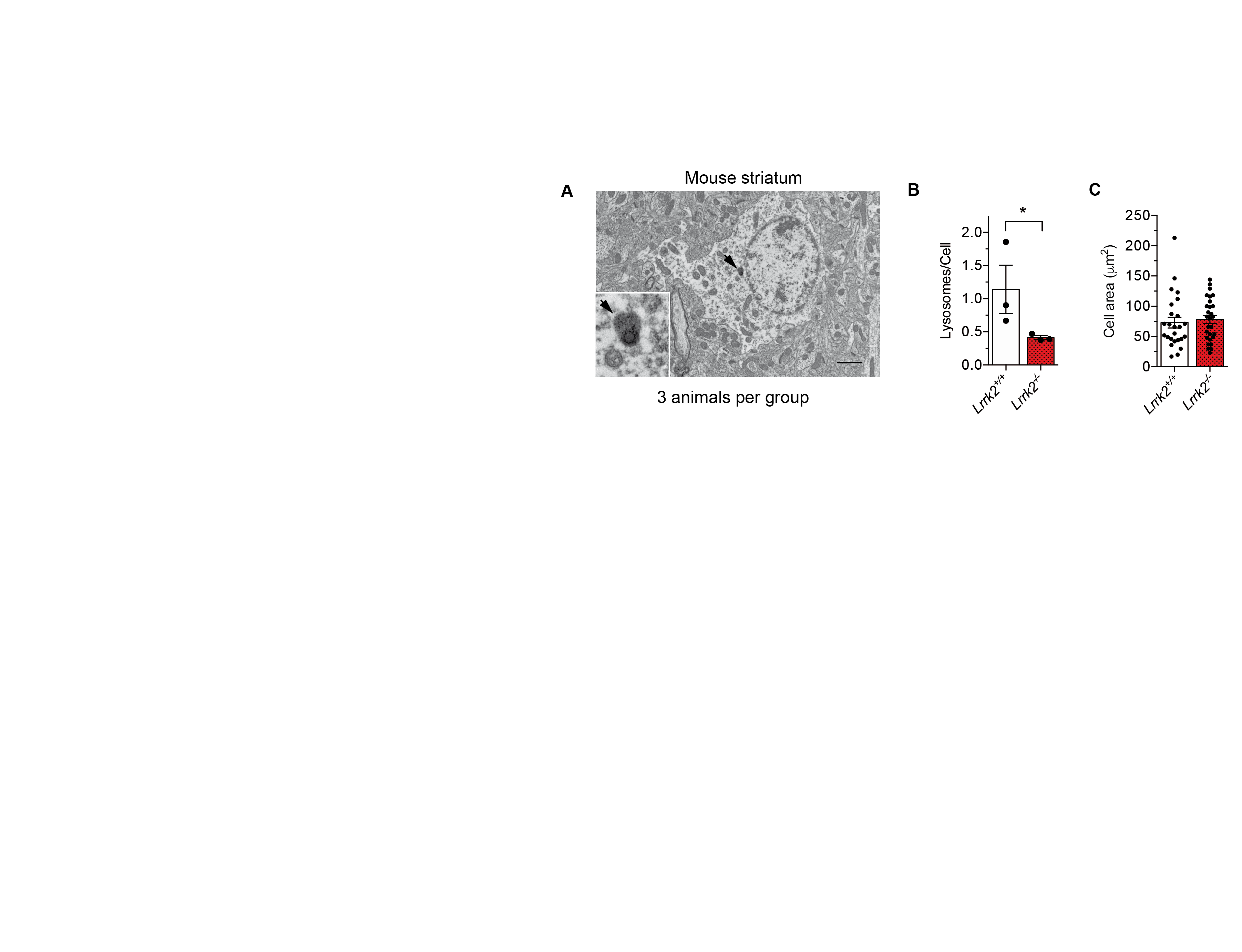
Supplementary Figure 7** Lrrk2^-/-^ astrocytes display less lysosomal-like structures in mouse striatal slices. **A)** Representative image of a Lrrk2 wild-type astrocyte containing a lysosome. **B)** Quantification of lysosomes in Lrrk2 wild-type and Lrrk2^-/-^ astrocytic cell bodies (Lrrk2^+/+^, n=3, 20 to 30 cells per animal; Lrrk2^-/-^, n=3, 15 to 20 cells per animal); p < 0.01, unpaired t-test). **C)** Quantification of astrocyte area; p > 0.05, unpaired t-test). Scale bar = 10 µm.


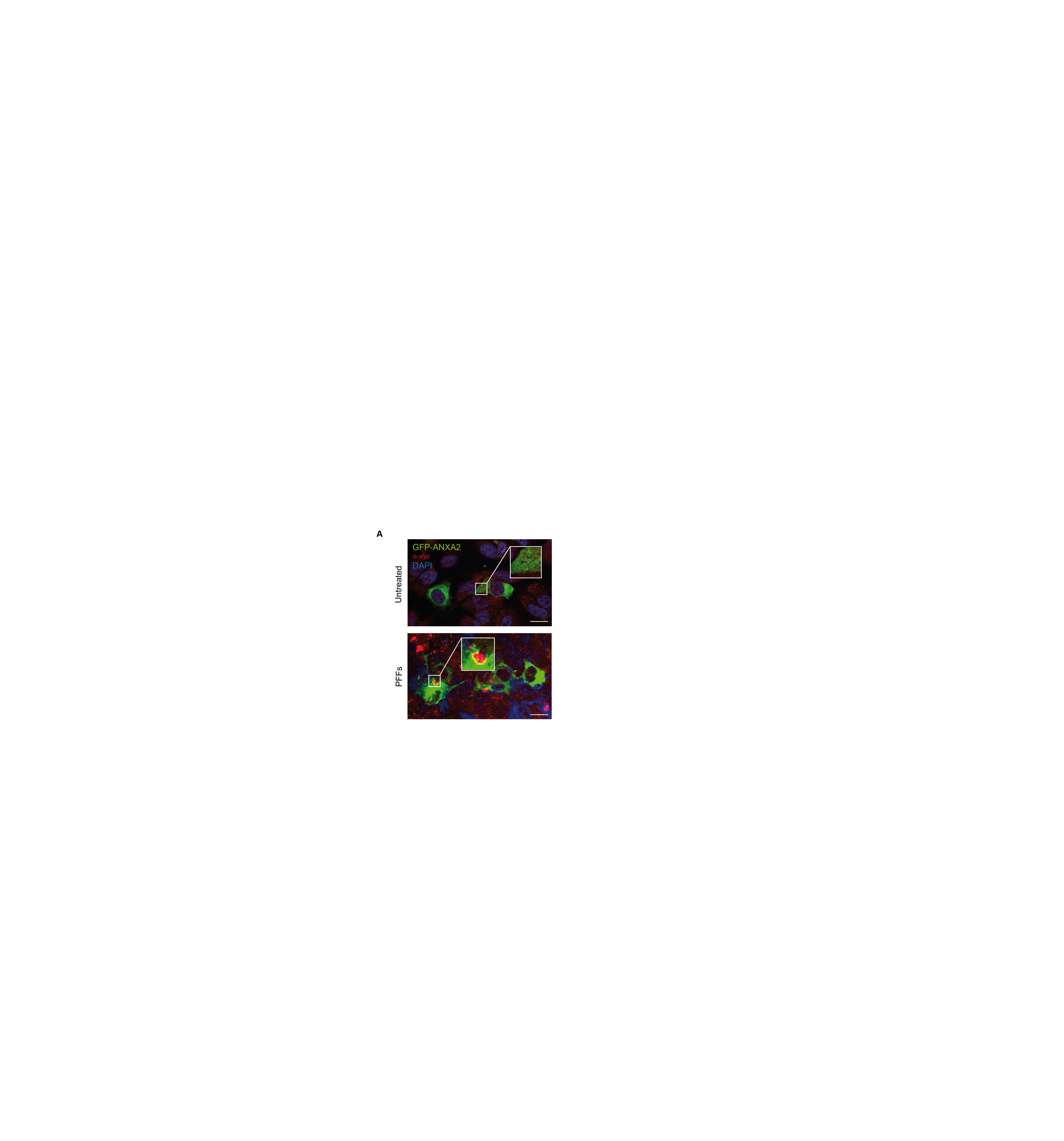


**Supplementary Figure 8** Representative images of H4 cells transfected with GFP-ANXA2 in PFF-treated and basal condition (GFP, green; DAPI, blue; α‑syn, red). Scale bars 20 µm.

**Supplementary Figure 9** Setting of AnxA2 downregulation in primary astrocytes. **A)**Western blot analysis of primary striatal astrocyte lysates transfected with scramble siRNA and AnxA2 siRNA using anti-Lrrk2, anti-β-actin and anti-AnxA2 antibodies. **B** and **C)**Quantification of band intensity was performed using ImageJ and normalized by β-actin (n=3). **D)** Immunocytochemistry of AnxA2 endogenous expression in scramble siRNA and AnxA2 siRNA transfected cells using anti-AnxA2 antibody**.** Recipient cells are visualized by GFP expression. **E)** Eight images were analyzed (n=2 independent cultures). Quantification of Anxa2 puncta was performed using ImageJ. Statistical analysis in **E** was made by Unpaired T-test * p ≤ 0.05 ** p ≤ 0.01 *** p ≤ 0.001. Scale bar = 30 µm

Supplementary Materials and Methods:

Transmission Electron Microscopy and image analysis in mouse brain slices

The preparation of TEM brain samples includes the staining with osmium tetroxide, resin embedding, and ultrathin sectioning of the brain sections (2). Briefly, sections from Lrrk2^-/-^ striatum were spread flat using a fine paintbrush before adding 1% osmium tetroxide in PBS for 30 minutes. Then, the sections will be dehydrated in increasing concentrations of ethanol in the following order: 35%, 35%, 50%, 70%, 80%, 90%, 100%, 100%, 100%. The dehydrated-sections were then immersed in propylene oxide and subsequently transfered from propylene oxide solution into a previously prepared plastic resin and maintained overnight for infiltration. The day after, sections were embedded with the resin on poly-chloro-tri-fluoro-ethylene (PCTFE) filmsheets and the resin polymerized in an incubator for 3 days at 55-60°C. Using a Leica UC7 ultramicrotome, 70-nm sections containing the dorsal striatum were then generated. Upon completion of these procedures, the material was ready for TEM examination. Astrocytes have pale nuclei, usually with a thin rim of heterochromatin beneath the nuclear envelope (https://www.bu.edu/agingbrain/chapter-11-astrocytes/). Occasionally, their cytoplasm is ‘watery’ and displays several inclusions and lipid accumulations (reactive state). In Figure 5A, we showed a rapresentative image of striatal astrocytes from wild-type animals. Images were acquired using a FEI Tecnai Spirit G2 TEM equipped with an ORCA-HR digital camera (10 MP; Hamamatsu) and operating at an accelerated voltage of 80 kV. A minimum of 20 astrocytic cell bodies per mouse were imaged randomly and analyzed for their area, with the experimenters blinded to genotypes during imaging and analysis. No differences in astrocyte area a were detected comparing Lrrk2^+/+^ versus Lrrk2^-/-^ mice. Conversely, the number of lysosomes in astrocytic cell bodies is decreased in the Lrrk2 knock-out animals (Lrrk2^+/+^, n=3 and Lrrk2^-/-^, n=3) (Figure A5).

1. Zhang Y, Chen K, Sloan SA, Bennett ML, Scholze AR, O'Keeffe S, Phatnani HP, Guarnieri P, Caneda C, Ruderisch N, et al: An RNA-sequencing transcriptome and splicing database of glia, neurons, and vascular cells of the cerebral cortex. J Neurosci 2014, 34:11929-11947.
2. J Vis Exp. 2016 Jun 1;(112). doi: 10.3791/54060. Correlative Light and Electron Microscopy to Study Microglial Interactions with β-Amyloid Plaques. Bisht K1, El Hajj H1, Savage JC1, Sánchez MG1, Tremblay MÈ2.
